# Reported Dietary Intake and Food Sources of Zinc, Selenium, and Vitamins A, E and C in the Spanish Population: Findings from the ANIBES Study †

**DOI:** 10.3390/nu9070697

**Published:** 2017-07-06

**Authors:** Josune Olza, Javier Aranceta-Bartrina, Marcela González-Gross, Rosa M. Ortega, Lluis Serra-Majem, Gregorio Varela-Moreiras, Ángel Gil

**Affiliations:** 1 Department of Biochemistry and Molecular Biology II, Institute of Nutrition and Food Sciences, University of Granada, Campus de la Salud, Avda. del Conocimiento, Armilla, 18016 Granada, Spain; jolza@ugr.es; 2 Instituto de Investigación Biosanitaria ibs.GRANADA, 18012 Granada, Spain; 3 CIBEROBN, Biomedical Research Networking Center for Physiopathology of Obesity and Nutrition, Carlos III Health Institute, 28029 Madrid, Spain; javieraranceta@gmail.com (J.A.-B.); marcela.gonzalez.gross@upm.es (M.G.-G.); lluis.serra@ulpgc.es (L.S.-M.); 4 Department of Food Science and Physiology, University of Navarra, c/Irunlarrea 1, 31008 Pamplona, Spain; 5 ImFINE Research Group, Department of Health and Human Performance, Universidad Politécnica de Madrid, c/Martín Fierro 7, 28040 Madrid, Spain; 6 Department of Nutrition, Faculty of Pharmacy, Madrid Complutense University, Plaza Ramón y Cajal s/n, 28040 Madrid, Spain; rortega@ucm.es; 7 Research Institute of Biomedical and Health Sciences, University of Las Palmas de Gran Canaria, Faculty of Health Science, c/Doctor Pasteur s/n Trasera del Hospital, Las Palmas de Gran Canaria, 35016 Las Palmas, Spain; 8 Spanish Nutrition Foundation (FEN), 28010 Madrid, Spain; gvarela@ceu.es or gvarela@fen.org.es; 9 Department of Pharmaceutical and Health Sciences, Faculty of Pharmacy, CEU San Pablo University, Urb. Montepríncipe, Crta. Boadilla Km 53, Boadilla del Monte, 28668 Madrid, Spain

**Keywords:** ANIBES study, trace elements, vitamins, misreporting, food intake

## Abstract

Zinc, selenium, and the vitamins A, E and C, all have specific biological functions that are involved mainly in the antioxidant defence system, which has important implications for the development of chronic diseases. We aimed to assess the reported intake of those six nutrients, as well as the food that contributes to their sources of intakes. Data were obtained from the Spanish ANIBES (“Anthropometry, Intake and Energy Balance in Spain”) study, *n* = 2009 (9–75 years old). The analyses were performed in the whole population and in the plausible energy reporters after a misreporting analysis according to the European Food and Safety Authority (EFSA) protocol. A validated, photo-based three-day food record was used to collect the data. Mean (max−min) reported intake for the whole population of zinc was 8.1 ± 0.1 mg/day, (2.3–27.3 mg/day), selenium 75 ± 1 µg/day, (14–265 µg/day), vitamin A 668 µg RE/day (2–11,017 µg RE/day), retinol 364 ± 18 µg/day (0–10,881 µg/day), carotenes 1735 ± 35 µg/day (13–13,962 µg/day), vitamin E 7.0 ± 0.1 mg α-TE/day (0.7–55.2 mg α-TE/day) and vitamin C 84.4 ± 1.4 mg/day (5.0–802.7 mg/day). The main source intakes for zinc were meat and meat products, for selenium cereals and grains, for vitamin E oils and fat, and for vitamin A and C vegetables. There is an elevated percentage of the Spanish ANIBES population not meeting the EFSA recommended intakes for all analysed micronutrients: zinc (83%), vitamin A (60%), vitamin E (80%), vitamin C (36%) and selenium (25%).

## 1. Introduction

In the last few decades, there has been an increase in the prevalence of nutrition-related non-communicable diseases, including obesity, cardiovascular diseases, and type 2 diabetes mellitus [1,2]. It has been suggested that this could be the result of a nutrition transition characterised by changes in the dietary pattern towards an unbalanced and unhealthy diet [3], accompanied by an unhealthy lifestyle that includes physical inactivity and sedentary behaviour [1]. 

Adequate nutrition is one of the pillars of public health, and knowing the population’s nutritional situation, before designing national guidelines, it is essential to improve the nutrition of the population [4]. Zinc, selenium, and vitamins A (retinol and carotenes), E and C, have in common biological functions involved in the antioxidant defence system, which have important implications for the prevention of inflammatory chronic diseases and in particular of cardiovascular illnesses.

Zinc is an essential trace element that participates in many metabolic processes as a catalytic, regulatory and structural component [5]. It is a cofactor for more than 300 enzymes and it is part of the structure of 2500 transcription factors [6]. It is also involved in the metabolic hormone regulation of growth and has key roles in gene expression regulation and the immune system. Selenium’s main biological role is associated with glutathione peroxidase (GPOX) and avoiding toxicity by selenoproteins [7]; apart from its antioxidant function, these proteins are involved in spermatogenesis, brain development, and thyroid function [7]. Vitamin A comprises retinol and the molecules that share its biological activity (retinoids), and those with provitamin A activity (carotenoids) [8]. Vitamin A participates in many biological functions such as the visual cycle, cell differentiation, cell proliferation and apoptosis, maintenance of epithelial tissue, reproduction and embryogenesis, haematopoiesis, intercellular communication, antioxidant defence, and immune competence [9]. Vitamin E is an effective antioxidant in the protection of unsaturated fatty acids and other easy oxidizable substances. This vitamin participates mainly in the stabilisation of biological membranes, the inhibition of platelet aggregation, the maintenance of the erythrocyte morphology and influences the activity of some enzymes [10]. Vitamin C is an antioxidant with a high reducing power. This vitamin participates as a cofactor in many biochemical reactions namely in the synthesis of collagen, carnitine, and catecholamines. It is also involved in the metabolism of cholesterol [11]. 

National diet survey, including a three-day food record, is the most common tool to evaluate the nutrient self-reported intake and the nutritional situation of the population. However, by using this kind of methodology, people tend to misreport their energy intake (EI), as it is mainly auto-reported [12]. Consequently, the reported EI does not represent the usual intake giving an estimate EI that is not physiologically plausible [12]. ANIBES (Anthropometry, Intake and Energy Balance in Spain) is a Spanish study that evaluates energy intake and expenditure, body composition and dietary patterns in a national representative sample. Previous articles have reported intake of energy [13], the main macronutrients [14] and several micronutrients [15,16]. As part of the representative Spanish ANIBES study [17], in the present article, we analysed the reported intake of zinc, selenium, and the vitamins A (retinol and carotenes), E, and C in the whole population, and in the plausible energy reporters separately (following EFSA harmonised approach to identify misreporting), and assessed the food that contributes to their sources of intake. 

## 2. Materials and Methods

The complete design, protocol, and methodology of the ANIBES study have been described in detail elsewhere [17]. 

### 2.1. Sample

The ANIBES is a cross-sectional study conducted using multistage stratified sampling. The sample for the ANIBES Study was designed based on 2012 census data published by the INE (Instituto Nacional de Estadística/Spanish Bureau of Statistics) for gender, age, habitat size and region [17]. The fieldwork was performed at 128 sampling points across Spain and the study was conducted from mid-September 2013 to mid-November 2013. The final sample comprised 2009 individuals aged 9–75 years (1013 men, 50.4%; 996 women, 49.6%) [17]. For the youngest (9–12, 13–17, and 18–24 years) and oldest (65–75 years) age groups, a “booster sample” to provide at least 200 individuals per age group (error ±6.9%) was included. Therefore, the random sample plus booster sample comprised 2285 participants. 

Subjects included in the study were those that were not on a prescribed diet; were following healthy lifestyle recommendations for the control or the prevention of diseases such as type 2 diabetes, hypertension, hypercholesterolemia, hypertriglyceridemia or hyperuricemia; individuals with food allergies or food intolerance and those diagnosed with metabolic diseases such as hyper or hypothyroidism. The subjects that were excluded from the study were those that were on a prescribed diet due to medical tests, pre- or post-surgery situation, diagnosed disease or any pathological or physiological situation or those with any disease or illness (e.g., cold, gastroenteritis, chicken pox, etc.)

The sample quotas according to the following variables were: age groups (9–12, 13–17, 18–64, and 65–75 years); sex (men/women); geographical distribution (Northeast, East, Southwest, North-Central, Barcelona, Madrid, Balearic and Canary Islands); and locality size: 2000 to 30,000 inhabitants (rural population), 30,000 to 200,000 inhabitants (semi-urban population) and over 200,000 inhabitants (urban population). Additionally, other factors, such as unemployment rate, the percentage of foreigners, physical activity level, and educational and economic level, were also considered [17,18].

The final protocol was approved by the Ethical Committee for Clinical Research of the Region of Madrid (Spain).

### 2.2. Food Record and Adequacy of Reported Intake

Study participants were provided with a tablet device (Samsung Galaxy Tab 2 7.0, Samsung Electronics, Suwon, South Korea). They recorded information, during two weekdays and one weekend day, before starting to eat and drink, and again after finishing. Additionally, a brief description of meals, recipes, brands, and other relevant information was registered using the tablet. Participants who declared or demonstrated that they were unable to use the tablet device were offered other options, such as using a digital camera, paper record or telephone interviews. In total 79% of the sample used a tablet, 12% a digital camera, and 9% a telephone interview. Food records were returned from the field in real time, to be coded by trained coders, supervised by dieticians. An ad hoc central server software/database was developed for this purpose to work in parallel with the coding and verification processes [17]. Food, beverage, and energy and nutrient reported intakes were calculated from food consumption records using VD-FEN 2.1 software, a Dietary Evaluation Program from the Spanish Nutrition Foundation (FEN). The program was newly developed for the ANIBES study by the FEN and is based mainly on Spanish food composition tables [19]. Data obtained from food manufacturers and nutritional information provided on food labels were also included. A food photographic atlas was used to assist in assigning gramme weights to portion sizes. 

Reported intake data were compared with national [20] and European [21] daily recommendations. The disparity between reported consumption and the level needed for adequacy was calculated comparing with 80% of the Spanish dietary reference value (DRV) [20] and EFSA population reference intake (PRI) or adequate intake (AI) [21].

### 2.3. Evaluation of Misreporting

In the present study, EFSA protocol to assess misreporting was used [22]. The methodology has been detailed somewhere else [15]. The procedure proposed by EFSA evaluates the reported energy intake (EIrep) against the presumed energy requirements. EIrep is expressed as a multiple of the mean basal metabolic rate estimated (BMRest), and it is compared with the presumed energy expenditure of the studied population. Subsequently, the ratio EIrep:BMRest is referred to as the physical activity levels (PAL) The PAL is established for young (≤17 years) and adults (≥18 years) in three levels, low 1.6 and 1.4; moderate 1.8 and 1.6; and vigorous 2.0 and 1.8, respectively. The protocol indicates that the analyses should be performed at group and individual levels. The group level determines the overall bias to the reported EI, and the individual level shows the rate of under and over reporters. To calculate the misreporting at both levels, the lower and upper cut-off values were specifically calculated for our population (Table 1). The BMRest was calculated using the Schoefield equations [23], and the physical activity was assessed during interviews with the international physical activity questionnaire (IPAQ) [24]. Misreporting cut-offs at group and individual levels for the ANIBES study are shown in Table 1. CV-WEI (coefficient of variance in energy intake within-subject) for the ANIBES population were 36.6% for children and adolescents and 41.6% for adults, respectively; and S (factor that considers the variation in energy intake, BMR and PAL) for children and adolescents was 27.3 and for adults 29.6. 

### 2.4. Statistical Analysis 

Data are expressed as mean ± standard error of the mean (SEM), median, ranges, percentiles and percentages. Normality was assessed using Kolmogorov–Smirnoff normality test for the random sample (2009 participants) and random + booster sample (2285). Appropriated non-parametric statistical tests were used for those variables that did not follow the normality. The random sample was used to show the total sample data and to compare between sexes. To compare by sex in each age group, the booster sample was included to enlarge those groups less represented in the random sample. Comparisons between groups were performed using a Student’s *t*-test for independent samples or Mann–Whitney U test to evaluate differences by sex within the whole population and within each age group. Analyses of variance (ANOVA) tests with Bonferroni correction for multiple comparisons or Kruskal–Wallis analysis was used to calculate differences among each age group [15]. These procedures have considered the sampling complexity during the stratification of the study design. The significance level was set at *p* < 0.05. Analyses were performed using IBM SPSS version 22.0 (IBM Corp., Armonk, NY, USA).

## 3. Results

### 3.1. Zinc, Selenium, and Vitamins C, A (Retinol and Carotenes), and E Intake in the Whole Population

Table 2 shows the daily reported intake levels of zinc, selenium, carotenes, retinol, vitamin A, E and C. Supplementary Tables S1–S7 show the percentiles distribution of each nutrient in the whole population and separately by age groups and sexes. 

Lower reported intake of zinc, selenium and vitamin E were observed in the elderly group compared with the other three age groups. Opposite to this, the reported intakes of carotenes and vitamin C increased with age. Likewise, intakes of zinc, selenium, retinol and vitamin E were higher in men than in women in the whole population, as well as for zinc in all age groups. Separately by age groups, the mean reported intake of selenium was higher in men than in women, in adolescents, adults and elderly groups, for retinol in children and adults and for vitamin E, only in adults. The reported intake of carotenes and vitamin C was lower in men than in women in the entire population. No differences were found for vitamin A.

### 3.2. Zinc, Selenium, Retinol, Carotenes, and Vitamins A, C and E Reported Intake in Plausible Energy Reporters

Table 3 shows the misreporting data. In the whole population, the plausible energy reporters were 543 individuals (27%) and the non-plausible energy reporters were 1466 (73%). The percentages of plausible energy reporters by age groups were: children 56%, adolescents 36%, adults 26% and elderly 22% [15]. The reported consumption of the studied nutrients was significantly higher (*p* < 0.05) in the plausible energy reporters than in the non-plausible energy reporters in the entire population, as well as divided by age group for all nutrients. When comparing the plausible and non-plausible energy reporters by sex, the reported intake of zinc, selenium, carotenes, and vitamins A, C and E were significantly different. 

### 3.3. Disparity between Reported Intake and the Level Needed for Adequacy for Zinc, Selenium, and Vitamins C, A (Retinol and Carotenes), and E in the Whole Population and in the Plausible Energy Reporters

Table 4 shows the percentage of the entire population and the plausible energy reporters that did not meet the 80% of the Spanish [20] and European [21] recommended daily intakes. As we can observe neither the whole population nor the plausible energy reporters met the daily intake recommendations for zinc, and vitamins A, E and C. Nevertheless, it is interesting to highlight that the inadequate intake of vitamin C in the elderly group was only 15% and 7% in the entire and plausible energy reports, respectively, according to the Spanish recommendations. In the case of selenium, children and adolescents showed an adequate intake, and only 11% of adults and 7% of elderly showed an inadequate intake according to Europe references. 

### 3.4. Contribution of the Food and Beverages to Zinc, Selenium, Retinol, Carotenes and Vitamins A, C and E Intakes

**Figure 1:**
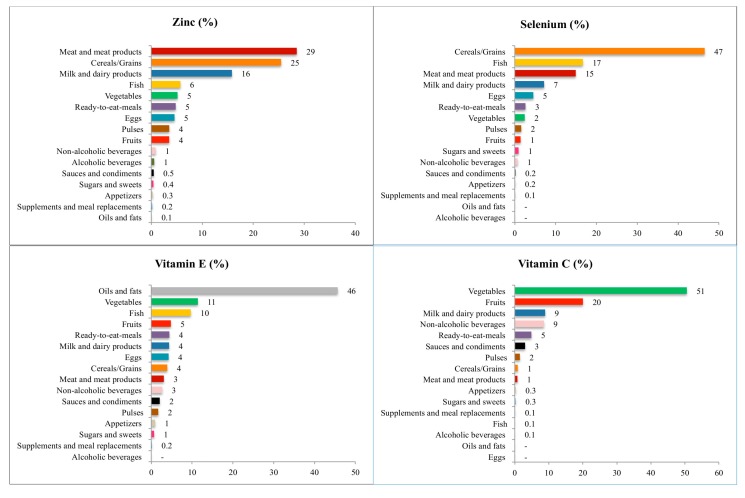
Contribution of food sources to the daily zinc, selenium, and vitamins E and C intake in the ANIBES Study population.

**Figure 2:**
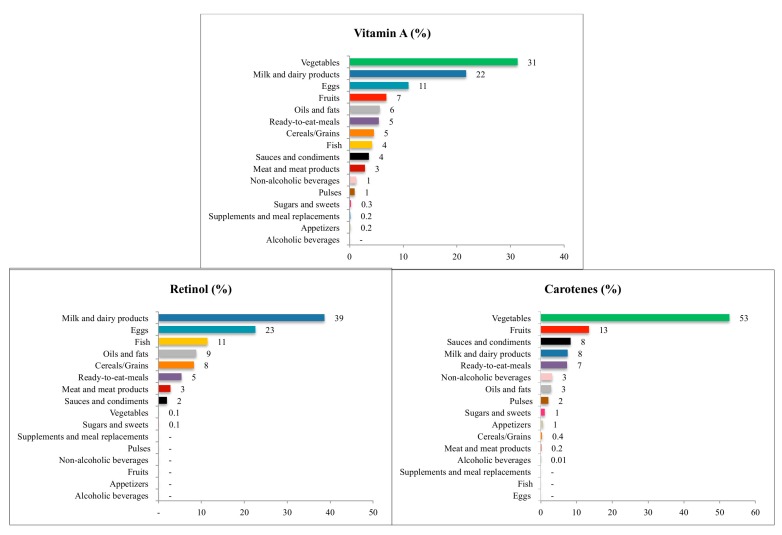
Contribution of food sources to the daily zinc, selenium, and vitamins E and C intake in the ANIBES Study population.

Figure 1 and Figure 2 show the contribution (%) of the food and beverage categories to daily zinc, selenium, vitamins E and C, retinol, carotenes and vitamin A intake for the entire population. Supplementary Tables S8–S14 show these data separately by age groups.

#### 3.4.1. Zinc

The main sources of zinc for the entire population were meat and meat products (28.5%; this contribution was lower in elderly, 24.7%), cereals and grains (25.5%), and milk and dairy products (15.8%). This last group provided higher percentages to the children. Fish (5.7%), vegetables (5.2%), and ready-to-eat meals (4.8%) complete the list to reach more than the 85% of the total intake of zinc. Fish and vegetables afforded a higher percentage to the older groups while ready-to-eat meals did so for, the younger groups. 

#### 3.4.2. Selenium

The largest source of selenium for the whole population was the group of cereals and grains (46.5%), with a higher contribution for the adolescents (50.8%). Fish provided 16.7%, meat and meat products 14.9%, and milk and dairy products 7.2%. Fish afforded a higher percentage to the older groups while meat and meat products and milk and dairy products contributed to a lower percentage only for the elderly group. All these groups afforded in more than 85% to the selenium intake.

#### 3.4.3. Vitamin E

Oil and fats were the main contributors (45.7%) to the vitamin E intake, followed by vegetables (11.4%), fish (9.7%), and fruits (4.8%). These three last food groups increased their contribution with age. Ready-to-eat meals, milk and dairy products, and eggs, contributed 4.4%, 4.4%, and 4.3%, respectively, to the intake of this vitamin. All these groups afforded in more than 85% to the vitamin E intake.

#### 3.4.4. Vitamin C

For the whole population, vegetables (50.6%) and fruits (20%) contributed to more than 70% to the intake of vitamin C. Milk and dairy products and non-alcoholic beverages ranked third and four, contributing in 8.9% and 8.7%, respectively. All these groups supplied more than 85% to the total vitamin C intake. These data reflect the vitamin C intake of the older groups. For the younger groups, vegetables were also the main contributors to the intake of vitamin C; however, for children, this food group represented 39.9%, second in the rank was fruits (15.2%), third was non-alcoholic beverages (15.7%) and fourth was milk and dairy products (14.7%). For adolescents, vegetables afforded 45.1%, milk and dairy products 13.3%, fruits 12.8% and non-alcoholic beverages 12.7%.

#### 3.4.5. Retinol

Milk and dairy products were the main source of retinol (38.7%) for the whole population, although it contributed much less to the older adults group. Eggs provided 22.6% and fish 11.4%, these last two food groups contributed in a higher proportion for the elderly group. Finally, oils and fats afforded 8.8% to complete the list that contributes more than 85% of the total daily retinol intake.

#### 3.4.6. Carotenes

Vegetables afforded more than half of carotenes intake for the entire population (52.7%), contributing to a much higher percentage for the older groups compared with the younger groups. Fruits ranked second (13.5%) and sauces and condiments third (8.4%); fruits provided more percentage to the elderly, while sauces and condiments did so to the younger groups. Milk and dairy products contributed 7.5% and ready-to-eat meals 7.4%; these last two food groups afforded more to the younger groups and less to the elderly. All these groups afforded more than 85% to the carotenes intake.

#### 3.4.7 Vitamin A

Vegetables were the main source of vitamin A for the whole population (31.3%), contributing in higher proportions in the older groups. Milk and dairy products provided 21.7% to the entire population, contributing more to the younger groups. Eggs ranked third (11%) and fruits fourth (6.9%); this last food group provided less among younger groups and much more to the elderly. Oils and fats supplied 5.6%, ready-to-eat meals 5.5%, and cereals and grains 4.5% to the vitamin A intake for the whole population; these last two food groups contributed more to the adolescents and less to the elderly. All these groups supplied more than 85% of the total vitamin A intake.

## 4. Discussion

Recent studies have demonstrated that a well-balanced diet leads to an improved redox status, which affects positively to reduce the risk of non-communicable chronic diseases [25]. The present article analyses the daily intake of the main micronutrients involved in the antioxidant defence system. Here, we show that the percentage of the Spanish population included in the ANIBES study not meeting the European recommendations for zinc, selenium, and vitamins A, E and C were 83%, 15%, 74%, 80% and 56%, respectively. Even when the plausible energy reporters were analysed separately, these percentages remained above 40%, except for selenium, where only 9% of the population showed inadequate intake.

In recent years, there has been controversy about the validity of the use of Memory-Based Dietary Assessment Methods (M-BMs), as these methods are indirect and have a pseudo-quantitative nature. Some authors believe that the data collected through them are inappropriate to calculate EI and have stated its inadmissibility in scientific research and for the formulation of national dietary guidelines, while other authors are opposed to these statements [26,27]. Additionally, sometimes the estimated EI of part of the population is not always plausible physiologically [12] because, for various reasons, people tend to underreport their food intake. To minimise all these methodological risks, the present study is based on the “Guidance on the EU Menu methodology” [28], a guidance document published by EFSA to facilitate the collection of harmonised food consumption data from all EU Member States. Additionally, some objective tools to measure EI were used, such as tablets and digital cameras. 

Spain has undergone dramatic socioeconomic changes since the 1960s. These changes include the increase in the immigrant population, the rural-urban migration, a rapid urbanisation process and the incorporation of women into the active workforce, factors that have influenced family life and home meals organisation. The increasing use of restaurants, catering and vending machines have also influenced food consumption. The Food Consumption Survey by the Ministry of Agriculture, Food and Environment (MAGRAMA), which has been conducted for over 20 years, evaluates the food consumption and dietary patterns in the Spanish population. The results of this survey have shown that the abovementioned social and economic changes have led to substantial modifications in food patterns in the last decades, moving the Spanish diet away from the traditional Mediterranean Diet pattern [29]. 

Comparing the data obtained in the present study with the data obtained in the ENALIA (Encuesta Nacional de ALimentación en Población Infantil y Adolescente de España/National Dietary Survey in Spanish Children and Adolescents) [30] for children and adolescents and with the ENIDE (Encuesta Nacional de Ingesta Dietética/National Dietary Intake Survey) [31] for adults, we observed that the zinc intake was similar for male children (9.8 mg/day), adolescents (11.3 mg/day) and adults (10–12 mg/day) as well as for women (8.7 mg/day, 8.9 mg/day and 8–9 mg/day, respectively) when we consider only the ANIBES plausible energy reporters. The intake of this nutrient is inadequate in the three studies when comparing with the Spanish and European recommendations. A recent review from Mensink et al. in nine European countries (Belgium, Denmark, France, Germany, The Netherlands, Poland, Serbia, Spain, and the UK) [32] reported that the intake of zinc in adolescents (11–17 years) ranged from 6.6 mg/day to 11.2 mg/day in girls, and from 8.4 mg/day to 14.7 mg/day in boys. The lowest values were observed in the UK while the highest were seen in Germany. Compared to the data obtained from the adolescents’ plausible energy reporters in the ANIBES study, both sexes reported intake was around the mean intake of the European countries included in the review. 

Studies in different countries of Europe have reported the consumption of Zn in adults. In men, Germany [33], Denmark [34], Finland [35,36], Italy [37] and Sweden [38] have reported mean intakes over 12 mg/day; Ireland [39] and The Netherlands [40] over 11 mg/day; and the United Kingdom (UK) [41] over 10 mg/day. In women, Finland [35,36] and Italy [37] have reported mean intakes over 10 mg/day; Germany [33], Denmark [34] and Sweden [38] over 9 mg/day; and Ireland [39], The Netherlands [40] and UK [41] less than 8.6 mg/day. Comparing with the ANIBES plausible energy reporters data, the reported intake for both men and women was a bit lower than the European mean intake.

As expected, meat and meat products were the main sources of zinc for the ANIBES population, as well as for the ENIDE study [31]. However, the other food groups’ contributions to the reported intake of zinc were in different order and proportions: in the ANIBES study, cereals and grains, milk and dairy products, fish and vegetables; and, in the ENIDE study [31], pulses and nuts, fish, eggs, milk and dairies and cereals. 

In all studied groups, the reported intake of selenium met almost the totality of the Spanish [20] as well as the European recommendations [21]. In children and adolescents (male and female), the reported selenium intake in the ENALIA study [30] was higher than in the ANIBES study, even taking into account only the plausible energy reporters. However, comparing these data with data collected in European countries [32], where the observed intake ranged from 29 mg/day for the Danish girls to 46 mg/day for the French boys, ANIBES reported intakes from adolescents (both sexes) was much higher in the total population (boys 85 mg/day and girls 71 mg/day) as well as in the plausible energy reporters (boys 102 mg/day and girls 84 mg/day). 

In the whole adult population, the reported selenium intakes in both men and women were higher in the ANIBES study than in the ENIDE study [31], and these differences increased when considering only the plausible energy reporters. The plausible energy reporter’s reported intake was inadequate in less than 7% in most of the age by sex groups according to the Spanish [20] and European recommendation [21]; only the older women groups had a higher percentage of inadequacy according to the European recommendations [21]. Results from the ANIBES study indicate that the reported intake of selenium in the whole population as well as in the plausible reporters separately, in adults and elderly (men and women) was higher than in some European Countries, namely Denmark [34], Finland [35,36], Italy [37], The Netherlands [40] and Sweden [38].

In contrast to the ENIDE study [31], where fish was the main dietary source of selenium, in ANIBES, cereals and grains ranked first, followed by fish, but with a large difference in proportion compared to the ENIDE study [31]. This disparity might be because the sampling of each study was made in different periods of the year. In the ANIBES study, meat and meat products and milk and dairy products ranked next, while, in the ENIDE study [31], the food groups contributing to the selenium intake after fish were meat and meat products, cereals and eggs.

The reported intakes of vitamin A in the present study were lower than the Spanish [20] and European [21] recommendations, in both the whole population and the plausible energy reporters. Comparing the children and adolescents (male and female) groups with the ENALIA study [30], the reported vitamin A intake was lower for ANIBES in both the whole population and plausible energy reporters separately. Data from EU countries [32] indicates that Spanish boys (528 µg RE/day) and girls (420 µg RE/day) (data from the EnKid study) [42] reported the lowest intake, while Poland for boys (1800 µg RE/day) and Germany for girls (1500 µg RE/day) reported the highest intakes. Comparing to the ANIBES study results, neither the whole population nor the plausible energy reporters’ data are higher than the mean observed intake from the European Countries.

In the adult group, the mean of the observed intake of vitamin A in the ENIDE study [31] was around 730 µg RE/day; adult mean intake was 747 µg RE/day for males and 723 µg RE/day for females. Data from the ANIBES study for the whole population were lower (668 µg RE/day) than the one reported in the ENIDE study [31]; however, taking the plausible energy reporter’s group alone, the mean intake (790 µg RE/day) was higher than the ENIDE study [31]. Adult vitamin A intake data from European countries indicate that Germany [43] and Poland [44] (both sexes) have the highest intake, over 1800 µg RE/day for men and over 1215 µg RE/day for women. Other countries such as France [45,46], the UK [47] and Denmark [48] reported intakes over 1000 µg RE/day for men and over 800 µg RE/day for women. Comparing these data with the data reported in the ANIBES study, the Spanish vitamin A intake in the adult’s population is much lower in the whole population as well as in the plausible energy reporters. Interestingly, the older adults whole population have vitamin A intakes similar to the adult group; however, considering only the plausible energy reporters, both men and women reported intakes over 1000 µg RE/day as in most European countries mentioned before. 

Unlike the ENIDE study [31], vegetables were the main source of vitamin A in the ANIBES study, preceding milk and dairy products, eggs, fruits and oil and fats. In the ENIDE study [31], eggs were the main source of vitamin A intake, followed by vegetables, milk and dairy products, fish and fruits. Even though the first three groups of food were the same in both studies, the proportions that represent each one is very different. 

The reported intakes of vitamin E in the ANIBES study for all studied groups in both the whole population and the plausible energy reporters were much lower than the Spanish [20] and European [21] recommendations. Comparing the observed data from the ENALIA study [30], children and adolescents reported intake was higher in that study than in the ANIBES study in the whole population; however, these differences narrowed when considering only the plausible energy reporters. Mensink and co-workers’ [32] review data indicate that the vitamin E intake in European adolescents ranged from 7.6 mg/day to 18.6 mg/day in boys, and from 6.4 mg/day to 16.4 mg/day in girls, with the lowest intakes observed in Spain (data from the EnKid study) [42], while the highest were seen in Germany. The ANIBES study data indicates that Spanish intakes are still low compared with the data observed in the rest of the European countries included in the review. 

The adult’s observed intake for vitamin E in the ENIDE study [31] was around 14 mg α-TE/day, whereas in the ANIBES study it was half of that value (7 mg α-TE/day) for the whole population and 9 mg α-TE/day for the plausible energy reporters. According to the ENIDE study [31], the adult population have an adequate intake of this nutrient. However, the data from the ANIBES study indicates that 80% of the whole adult population and 60% of the plausible energy reporters have inadequate intake of vitamin E. The European adult’s intake of vitamin E data indicates that Germany [33] and The Netherlands [40] have the highest observed intake for men (over 14.5 mg α-TE/day) and women (over 11.5 mg α-TE/day); followed by Italy [37] and Ireland [39] whose observed intakes were over 11 mg α-TE/day for men and women. Countries such as Portugal [49], Sweden [38] and Denmark [50] have intakes around 8 mg α-TE/day for men and around 6 mg α-TE/day for women. In the ANIBES study, the adult’s whole population reported an intake of around 7 mg α-TE/day, the lowest among the European countries analysed here. However, taking into account just the plausible energy reporters, the reported intake is above 11 mg α-TE/day for men and almost 9 mg α-TE/day for women.

The main source of intake of vitamin E in both ENIDE [31] and ANIBES studies were oil and fats; however, in the ENIDE study [31], pulses, seeds and nuts ranked second, preceding fish, vegetables, eggs and fruits, which is very different from the ANIBES study, where vegetables ranked second, followed by fish, ready-to-eat-meals, and milk and dairy products and eggs.

The mean reported intakes for vitamin C in the ANIBES study was lower than the observed intakes in the ENALIA study [30] for children and adolescents, and the ENIDE study [31] for adults in the whole population and the plausible energy reporters separately. Data from the ENALIA study [30] indicate that only a subgroup of female adolescents had an inadequate intake of vitamin C; however, in the ANIBES study, both children and adolescents of both sexes did not meet the Spanish or European recommendations. In the ENIDE study [31], 100% of the adult population had an adequate intake of this vitamin. However, in the ANIBES study when we calculated the inadequate intake, we observed that 29% and 58% of the whole adult population did not meet the Spanish and European recommendations, respectively. Even considering only the plausible energy reporters, the recommendations were not met (19% and 45%). 

In their review of the EU countries, Mensink et al. [32] indicated that the vitamin C intake in adolescent boys ranged from 71 mg/day in The Netherlands to 203 mg/day in Germany, and in girls, from 69 mg/day in Spain (data from the EnKid study) [42] to 201 mg/day in Germany. They comment that the Germany reported data doubled the data from any other country. Even taking into account the previous comment, ANIBES reported data was lower than those reported for the lowest intake, considering only the plausible energy reporters, the reported intakes are still lower.

Many European countries have reported the mean intake of vitamin C in adults. Germany [33], Greece [49] and Norway [49] have reported intakes over 140 mg/day in men and women; Denmark [34], Portugal [49], Italy [37], Ireland [39] (men and women) and Finland (women) [35,36] between 100 and 130 mg/day; and Finland (men) [35,36], Sweden [38] and UK [41] (men and women) under 100 mg/day. The ANIBES reported intake of vitamin C for the adult’s whole population was around 84 mg/day and considering only the plausible energy reporters the intake was around 100 mg/day, both amounts lower than most of the European observed data. 

Fruits and vegetables are the main source of vitamin C (70%) in the ENIDE study [31] as it is in the ANIBES study albeit in the opposite order. In the ENIDE study [31], fruits and vegetables were followed by non-dairy drinks and pulses and in the ANIBES study by milk and dairy products, and non-alcoholic beverages.

As discussed in previously published articles, the ANIBES study has several strengths, which include the careful design, protocol, and methodology used, conducted among a random representative sample of the Spanish population aged 9–75 years [14,15,16,17,18,19,20,21,22,23,24,25,26,27,28]. It is the first Spanish study at national level that analysed the data for the whole population and the plausible energy reporters. The limitations of this study are its cross-sectional design, which provides evidence for associations but not causal relationships [24] and the low retention data found after the analysis of misreporting. When we comparing our findings with other studies, we observed that a low percentage of the energy intake studies apply the misreporting methodology and among these studies, the variability of the underreporting is extensive. In studies that have used the 24-h recall method, misreporting goes from 4% to 67%, and in studies that have used the food record method, it goes from 8% to 49% [51,52]. The usual range of misreporting seen in studies goes from 20% to 30%. As we can observe, our study shows a higher percentage of misreporting compared with most of the published studies on that issue. However, it is important to highlight two points: (1) Even with a lower total population that reported a plausible energy intake, we can assure that our data are reliable and reflect the true nutrient intake situation of the Spanish population. (2) Many studies about energy and nutrient intake, apart from national surveys, have an N lower than 500; in this respect, our N of plausible reporters is not negligible.

## 5. Conclusions

The reported intake of zinc and the vitamins A and E are low in the ANIBES population. In the whole studied group, 92% and 83% for zinc, 74% and 60% for vitamin A, and 80% and 80% for vitamin E, of the population had reported intakes below 80% of the Spanish and European recommended daily intakes, respectively; even when the plausible energy reporters, whose reported intakes were higher than the whole population, were analysed separately. For vitamin C, 29% and 56% of the population had reported intakes below 80% of the Spanish and European recommended daily intakes, respectively, but, separately by age groups, 7% and 20% of the older plausible energy reporters had reported intakes below 80%, respectively. For selenium, only 15% and 25% of the population had reported intakes below 80% of the Spanish and European recommended daily intakes respectively.

The main food source intakes for zinc were meat and meat products; for selenium were cereals and grains; for vitamin E oils and fat; and for vitamins A and C vegetables. A significant percentage of the Spanish ANIBES population does not meet the recommended intakes for zinc, vitamin A and vitamin E; a reasonable percentage of people does not meet the recommendations of vitamin C; and a low percentage of people does not meet the selenium recommendations.

## Figures and Tables

**Table 1:** Calculated misreporting cut-off at group and individual levels for the ANIBES study.

	Misreporting Cut-Off			
	Group Level		Individual Level	
PAL	Lower	Upper	Lower	Upper
Children and adolescents				
1.6	1.55	1.66	0.93	2.76
1.8	1.73	1.86	1.04	3.10
2.0	1.93	2.07	1.16	3.45
Adults and elderly				
1.4	1.38	1.42	0.77	2.53
1.6	1.58	1.62	0.88	2.89
1.8	1.77	2.83	1.00	3.25

PAL: Physical activity level. The PAL is established for children and adolescents; and adults and elderly in three levels, low 1.6 and 1.4; moderate 1.8 and 1.6; and vigorous 2.0 and 1.8, respectively.

**Table 2:** Daily zinc, selenium, vitamin A, retinol, carotenes, vitamin E and vitamin C reported intake by sex and age group in the ANIBES Study population.

	*Total*			*Children 9–12 Years*			*Adolescents 13–17 Years*			*Adults 18–64 Years*			*Elderly 65–75 Years*		
	*n*	Mean ± SEM	Median (Range)	*n*	Mean ± SEM	Median (Range)	*n*	Mean ± SEM	Median (Range)	*n*	Mean ± SEM	Median (Range)	*n*	Mean ± SEM	Median (Range)
**ZINC (mg/day)**															
**Total**	2009	8.1 ± 0.1	7.7 (2.3–27.3)	213	8.3 ± 0.1 ^a^	8.2 (3.7–17.3)	211	8.6 ± 0.2 ^a^	8.3 (2.9–18.6)	1655	8.2 ± 0.1 ^a^	7.7 (2.3–27.3)	206	7.4 ± 0.2 ^b^	7.1 (3.1–20.0)
* Men*	1013	8.8 ± 0.1 *	8.4 (2.3–27.3)	126	8.6 ± 0.2 *	8.2 (3.7–17.3)	137	9.2 ± 0.2 *	8.7 (2.9–18.6)	798	8.9 ± 0.1 *	8.6 (2.3–27.3)	99	8.1 ± 0.3 *	7.5 (3.7–20.0)
* Women*	996	7.4 ± 0.1	7.2 (2.9–19.5)	87	7.8 ± 0.2	7.6 (4.3–12.5)	74	7.4 ± 0.3	7.5 (3.6–13.5)	857	7.5 ± 0.1	7.2 (2.9–19.5)	107	6.8 ± 0.2	6.8 (3.1–12.3)
**SELENIUM (µg/day)**															
**Total**	2009	75 ± 1	72 (14–265)	213	77 ± 2 ^a^	76 (9–180)	211	80 ± 2 ^a^	77 (26–164)	1655	76 ± 1 ^a^	72 (14–265)	206	70 ± 2 ^b^	65 (23–221)
* Men*	1013	81 ± 1 *	77 (20–188)	126	79 ± 2	76 (9–180)	137	85 ± 2 *	81 (26–164)	*798*	82 ± 1 *	79 (20–198)	99	75 ± 3 *	70 (28–221)
* Women*	996	69 ± 1	67 (14–265)	87	74 ± 2	75 (24–140)	74	71 ± 3	67 (30–150)	*857*	70 ± 1	67 (134–265)	107	64 ± 2	61 (23–144)
**VITAMIN A (µg RE/day)**															
**Total**	2009	668 ± 19	477 (2–11,017)	213	664 ± 43	496 (79–5991)	211	570 ± 33	426 (108–3434)	1655	672 ± 21	479 (2–11,017)	206	658 ± 61	489 (78–7796)
* Men*	1013	691 ± 29	478 (38–11,017)	126	702 ± 51	531 (79–3196)	137	582 ± 42	446 (109–3434)	798	697 ± 34	484 (38–11,017)	99	708 ± 104	475 (96–7796)
* Women*	996	644 ± 24	474 (2–7505)	87	609 ± 75	427 (120–5991)	74	546 ± 53	383 (108–2831)	857	650 ± 26	474 (2–7505)	107	612 ± 67	492 (78–6584)
**RETINOL (µg/day)**															
**Total**	2009	364 ± 18	187 (0–10,881)	213	420 ± 42 ^a^	227 (18–5950)	211	343 ± 29 ^a^	218 (0–2697)	1655	363 ± 20 ^b^	186 (0–10,881)	206	309 ± 57 ^c^	163 (3–7407)
* Men*	1013	399 ± 28 *	199 (0–10,881)	126	461 ± 50 *	262 (18–2802)	137	359 ± 37	237 (0–2697)	798	395 ± 33 *	197 (0–10,881)	99	361 ± 98	167 (3–7407)
* Women*	996	327 ± 23	176 (0–7440)	87	362 ± 75	211 (46–5950)	74	312 ± 45	204 (21–2392)	857	333 ± 25	177 (0–7440)	107	261 ± 61	160 (9–6494)
**CAROTENES (µg/day)**															
**Total**	2009	1735 ± 35	1342 (13–13,962)	213	1331 ± 78 ^a^	995 (42–6222)	211	1254 ± 79 ^a^	882 (45–6805)	1655	1760 ± 39 ^b^	1355 (13–13,962)	206	2082 ± 122 ^c^	1618 (97–11,643)
* Men*	1013	1652 ± 46 *	1231 (14–10,960)	126	1283 ± 102	980 (50–5754)	137	1227 ± 100	873 (79–6197)	798	1696 ± 54	1313 (14–10,960)	99	2068 ± 151	1705 (123–6851)
* Women*	996	1820 ± 51	1415 (13–13,962)	87	1402 ± 121	1023 (42–6222)	74	1303 ± 132	993 (45–6805)	857	1819 ± 56	1419 (13–13,962)	107	2095 ± 189	1528 (97–11,643)
**VITAMIN E (mg α-TE/day)**															
**Total**	2009	7.0 ± 0.1	6.3 (0.7–55.2)	213	7.4 ± 0.3 ^a^	6.3 (0.7–27.6)	211	7.5 ± 0.3 ^a^	6.4 (1.1–31.0)	1655	7.1 ± 0.1 ^a^	6.5 (0.7–55.2)	206	5.9 ± 0.2 ^b^	5.2 (1.7–16.6)
* Men*	1013	7.3 ± 0.1 *	6.5 (0.7–55.2)	126	7.4 ± 0.4	6.1 (0.7–27.6)	137	7.6 ± 0.4	6.5 (1.1–24.0)	798	7.4 ± 0.2 *	6.7 (0.9–55.2)	99	6.3 ± 0.3	5.8 (1.8–16.6)
* Women*	996	6.7 ± 0.1	6.1 (0.7–27.5)	87	7.5 ± 0.4	6.6 (2.2–19.1)	74	7.4 ± 0.6	6.3 (1.7–31.0)	857	6.8 ± 0.1	6.3 (0.7–27.5)	107	5.6 ± 0.3	4.9 (1.7–15.7)
**VITAMIN C (mg/day)**															
**Total**	2009	84.4 ± 1.4	71.3 (5.0–802.7)	213	66.4 ± 3.2 ^a^	57.2 (6.9–258.3)	211	61.6 ± 3.1 ^a^	49.3 (4.5–270.5)	1655	84.8 ± 1.5 ^b^	71.8 (5.0–802.7)	206	106.6 ± 4.8 ^c^	94.6 (14.5–478.8)
* Men*	1013	83.2 ± 2.0 *	68.9 (5.0–802.7)	126	65.1 ± 3.7	56.6 (6.9–210.6)	137	62.6 ± 4.1	48.3 (4.5–270.5)	798	85.2 ± 2.3	72.0 (5.0–802.7)	99	109.4 ± 7.3	96.6 (16.2–410.6)
* Women*	996	85.6 ± 1.9	72.8 (8.0–788.6)	87	68.3 ± 5.5	58.5 (11.8–258.3)	74	59.9 ± 4.7	50.6 (8.8–234.3)	857	84.5 ± 2.0	71.8 (5.9–788.6)	107	104.1 ± 6.2	91.6 (14.5–478.8)

Results are expressed as the mean ± standard error of the mean (SEM) and median with range (in brackets); (*) *t*-test or Mann–Whitney U test was used to evaluate differences by sex within the whole population and within each age group. ANOVA or Kruskal–Wallis tests was used to calculate differences among age groups (mean values within the same row with unlike superscript letters were significantly different). *p* < 0.05 was considered statistically significant.

**Table 3:** Daily zinc, selenium, vitamin A, retinol, carotenes, vitamin E and vitamin C reported intake by plausible energy reporters, non-plausible energy reporters and age group in the ANIBES Study population.

	*Total*			*Children 9–12 Years*			*Adolescents 13–17 Years*			*Adults 18–64 Years*			*Elderly 65–75 Years*		
	*n*	Mean ± SEM	Median (Range)	*n*	Mean ± SEM	Median (Range)	*n*	Mean ± SEM	Median (Range)	*n*	Mean ± SEM	Median (Range)	*n*	Mean ± SEM	Median (Range)
**ZINC (mg/day)**															
**Total**	2009	8.1 ± 0.1	7.7 (2.3–27.3)	213	8.3 ± 0.2	8.2 (3.7–17.3)	211	8.6 ± 0.2	8.3 (2.9–18.6)	1655	8.2 ± 0.1	7.7 (2.3–27.3)	206	7.4 ± 0.2	7.1 (3.1–20.0)
*Plausible energy reporters*	543	9.8 ± 0.1 *	9.5 (4.9–23.0)	120	9.0 ± 0.2 *	9.0 (5.4–14.5)	76	10.3 ± 0.3 *	9.9 (4.9–16.7)	433	10.0 ± 0.1 *	9.7 (5.2–23.0)	45	9.5 ± 0.4 *	9.1 (5.4–20.0)
* Men*	232	11.0 ± 0.2 ^§^	10.7 (5.4–23.0)	68	9.4 ± 0.2	9.1 (6.5–14.5)	48	11.0 ± 0.3	10.9 (7.2–16.7)	158	11.6 ± 0.2	11.3 (5.8–23.0)	24	10.2 ± 0.7	9.6 (5.4–20.0)
* Women*	311	8.9 ± 0.1 ^†^	8.6 (4.9–19.5)	52	8.6 ± 0.3	8.8 (5.4–12.5)	28	9.0 ± 0.4	8.7 (4.9–13.5)	275	9.0 ± 0.1	8.7 (5.2–19.5)	21	8.7 ± 0.4	8.4 (6.3–12.3)
*Non-Plausible energy reporters*	1466	7.5 ± 0.1	7.1 (2.3–27.3)	93	7.3 ± 0.2	7.1 (3.7–17.3)	135	7.6 ± 0.2	7.6 (2.9–18.6)	1222	7.5 ± 0.1	7.2 (2.3–27.3)	161	6.9 ± 0.2	6.74 (3.1–17.8)
* Men*	781	8.2 ± 0.1	7.8 (2.3–27.3)	58	7.6 ± 0.3	7.3 (3.7–17.3)	89	8.2 ± 0.2	8.0 (2.9–18.6)	640	8.2 ± 0.1	7.9 (2.3–27.3)	75	7.4 ± 0.3	7.1 (3.7–17.8)
* Women*	685	6.7 ± 0.1	6.6 (2.9–19.2)	35	6.7 ± 0.3	6.6 (4.3–11.6)	46	6.5 ± 0.2	6.2 (3.6–9.8)	582	6.7 ± 0.1	6.6 (2.9–19.2)	86	6.4 ± 0.2	6.4 (3.1–11.2)
**SELENIUM (µg/day)**															
**Total**	2009	75 ± 1	72 (14–265)	213	77 ± 2	76 (9–180)	211	80 ± 2	77 (26–164)	1655	76 ± 1	72 (14–265)	206	70 ± 2	65 (23–221)
*Plausible energy reporters*	543	90 ± 1 *	87 (25–265)	120	83 ± 2 *	85 (39–180)	76	96 ± 3 *	91 (46–164)	433	91 ± 2 *	87 (25–265)	45	93 ± 4 *	85 (38–221)
* Men*	232	101 ± 2 ^§^	95 (31–188)	68	85 ± 3	86 (39–180)	48	102 ± 4	98 (64–164)	158	104 ± 2	100 (31–196)	24	101 ± 7	96 (49–221)
* Women*	311	82 ± 2 ^†^	78 (25–265)	52	81 ± 3	79 (39–140)	28	84 ± 5	79 (46–150)	275	83 ± 2	87 (25–265)	21	84 ± 5	83 (38–144)
*Non-Plausible energy reporters*	1466	70 ± 1	67 (14–185)	93	69 ± 2	64 (9–145)	135	71 ± 2	68 (26–147)	1222	70 ± 1	67 (14–172)	161	63 ± 2	61 (23–185)
* Men*	781	79 ± 1	71 (20–185)	58	72 ± 4	63 (9–145)	89	75 ± 3	72 (26–147)	640	77 ± 1	73 (20–172)	75	67 ± 3	63 (28–185)
* Women*	685	63 ± 1	60 (14–166)	35	65 ± 4	67 (24–99)	46	64 ± 4	57 (30–139)	582	63 ± 1	60 (14–166)	86	59 ± 2	58 (23–117)
**VITAMIN A (µg RE/day)**															
**Total**	2009	668 ± 19	477 (2–11,017)	213	664 ± 43	496 (79–5991)	211	570 ± 33	426 (108–3434)	1655	672 ± 21	479 (2–11,017)	206	658 ± 61	489 (78–7796)
*Plausible energy reporters*	543	790 ± 31 *	609 (145–7796)	120	724 ± 62 *	576 (79–5991)	76	685 ± 59 *	567 (156–3434)	433	779 ± 30 *	611 (92–5864)	45	1124 ± 209 *	717 (173–7796)
* Men*	232	860 ± 56 ^§^	626 (145–7796)	68	756 ± 66	589 (79–2814)	48	709 ± 74	567 (156–3434)	158	866 ± 63	639 (146–5864)	24	1133 ± 304	712 (268–7796)
* Women*	311	737 ± 34 ^†^	600 (147–6584)	52	681 ± 115	553 (143–5991)	28	644 ± 100	563 (232–2831)	275	729 ± 31	600 (92–3925)	21	1115 ± 290	732 (173–6584)
*Non-Plausible energy reporters*	1466	622 ± 23	425 (2–11,017)	93	587 ± 58	384 (86–3196)	135	504 ± 38	356 (108–2784)	1222	635 ± 27	431 (2–11,017)	161	527 ± 47	420 (78–6887)
* Men*	781	641 ± 34	431 (38–11,017)	58	638 ± 80	421 (86–3196)	89	514 ± 49	375 (109–2728)	640	655 ± 39	451 (38–11,017)	75	571 ± 93	415 (96–6887)
* Women*	685	601 ± 31	414 (2–7505)	35	502 ± 77	364 (120–2676)	46	489 ± 58	348 (108–2027)	582	613 ± 36	413 (2–7505)	86	489 ± 35	429 (78–1735)
**RETINOL (µg/day)**															
**Total**	2009	364 ± 18	187 (0–10,881)	213	420 ± 42	227 (18–5959)	211	343 ± 29	218 (0–2697)	1655	363 ± 20	186 (0–10,881)	206	309 ± 57	163 (3–7407)
*Plausible energy reporters*	543	423 ± 29 *	258 (21–7407)	120	451 ± 62 *	263 (38–5959)	76	422 ± 51 *	312 (54–2697)	433	405 ± 27 *	259 (21–5249)	45	597 ± 212 *	248 (62–7407)
* Men*	232	491 ± 53 ^§^	285 (48–7407)	68	483±63	278 (38–2594)	48	433 ± 62	340 (80–2697)	158	467 ± 57	282 (48–5246)	24	633 ± 306	252 (65–7407)
* Women*	311	372 ± 31 ^†^	225 (21–6494)	52	409 ± 116	217 (91–5950)	28	403 ± 90	227 (54–2392)	275	369 ± 27	235 (21–3585)	21	555 ± 298	228 (62–6494)
*Non-Plausible energy reporters*	1466	341 ± 22	166 (0–10,881)	93	381 ± 56	191 (18–2802)	135	298 ± 34	187 (0–2672)	1222	348 ± 26	168 (0–10,881)	161	228 ± 41	143 (3–6242)
* Men*	781	372 ± 33	178 (0–10,881)	58	434 ± 78	214 (18–2802)	89	320 ± 46	199 (0–2672)	640	378 ± 38	182 (0–10,881)	75	274 ± 85	147 (3–6242)
* Women*	685	307 ± 30	149 (0–7440)	35	293 ± 73	167 (46–2503)	46	256 ± 47	169 (21–1726)	582	315 ± 34	150 (0–7440)	86	189 ± 20	141 (9–1055)
**CAROTENES (µg/day)**															
**Total**	2009	1735 ± 35	1342 (13–13,962)	213	1331 ± 78	995 (42–6222)	211	1254 ± 79	882 (45–6805)	1655	1760 ± 39	1355 (13–13,962)	206	2082 ± 122	1618 (97–11,643)
*Plausible energy reporters*	543	2080 ± 75 *	1644 (65–13,159)	120	1472 ± 109 *	1094 (145–6222)	76	1468 ± 141 *	1013 (45–5676)	433	2119 ± 84 *	1685 (65–13,159)	45	3111 ± 348 *	2574 (339–11,643)
* Men*	232	2077 ± 118 ^§^	1601 (101–9795)	68	1419 ± 144	1053 (145–5754)	48	1561 ± 199	961 (101–5676)	158	2250 ± 150	1754 (124–9795)	24	2857 ± 361	2443 (687–6851)
* Women*	311	2083 ± 98 ^†^	1678 (65–13,159)	52	1542 ± 167	1122 (198–6222)	28	1309 ± 174	1042 (45–3695)	275	2044 ± 99	1649 (65–13,159)	21	3402 ± 625	2659 (339–11,643)
*Non-Plausible energy reporters*	1466	1607 ± 38	1237 (13–13,962)	93	1149 ± 108	831 (42–4665)	135	1133 ± 94	800 (62–6805)	1222	1633 ± 43	1264 (13–13,962)	161	1794 ± 113	1396 (97–8292)
* Men*	781	1525 ± 48	1165 (14–10,960)	58	1122 ± 143	810 (50–4665)	89	1047 ± 105	777 (79–6197)	640	1559 ± 54	1200 (14–10,960)	75	1815 ± 153	1550 (123–6517)
* Women*	685	1700 ± 59	1339 (13–13,962)	35	1194 ± 165	960 (42–4656)	46	1299 ± 185	864 (62–6805)	582	1713 ± 67	1342 (13–13,962)	86	1776 ± 164	1316 (97–8292)
**VITAMIN E (mg α** **-TE/day)**															
**Total**	2009	7.0 ± 0.1	6.3 (0.7–55.2)	213	7.4 ± 0.3	6.3 (0.7–27.6)	211	7.5 ± 0.3	6.4 (1.1–31.0)	1655	7.1 ± 0.1	6.4 (0.7–55.2)	206	5.9 ± 0.2	5.25 (1.73–16.59)
*Plausible energy reporters*	543	9.0 ± 0.2 *	8.3 (1.7–27.6)	120	8.3 ± 0.4 *	7.6 (2.0–27.6)	76	9.7 ± 0.6 *	9.0 (1.7–31.0)	433	9.2 ± 0.2 *	8.2 (2.2–27.5)	45	8.3 ± 0.4 *	8.21 (3.90–15.70)
* Men*	232	9.8 ± 0.3 ^§^	9.0 (3.2–27.6)	68	8.3 ± 0.5	7.2 (1.9–27.6)	48	9.8 ± 0.7	9.2 (3.9–24.0)	158	10.2 ± 0.3	9.4 (3.2–27.3)	24	8.5 ± 0.5	8.4 (4.4–15.4)
* Women*	311	8.5 ± 0.2 ^†^	7.9 (1.7–27.6)	52	8.4 ± 0.6	7.7 (2.5–19.1)	28	9.6 ± 1.2	8.4 (1.7–31.0)	275	8.7 ± 0.2	8.0 (2.2–27.5)	21	8.0 ± 0.7	8.2 (3.9–15.7)
*Non-Plausible energy reporters*	1466	6.3 ± 0.1	5.7 (0.7–55.2)	93	6.2 ± 0.3	5.5 (0.7–18.7)	135	6.3 ± 0.3	5.4 (1.1–20.5)	1222	6.4 ± 0.1	5.9 (0.7–55.2)	161	5.3 ± 0.2	4.81 (1.73–16.59)
* Men*	781	6.6 ± 0.1	6.0 (0.7–55.2)	58	6.3 ± 0.4	5.6 (0.7–18.7)	89	6.4 ± 0.4	5.5 (1.1–20.5)	640	6.7 ± 0.2	6.1 (0.9–55.2)	75	5.6 ± 0.3	5.0 (1.8–16.6)
* Women*	685	5.9 ± 0.1	5.4 (0.7–23.3)	35	6.1 ± 0.6	5.0 (2.2–18.5)	46	6.1 ± 0.5	5.4 (2.3–14.3)	582	6.0 ± 0.1	5.8 (0.7–23.3)	86	5.0 ± 0.2	4.7 (1.7–14.6)
**VITAMIN C (mg/day)**															
**Total**	2009	84.4 ± 1.4	71.3 (5.0–802.7)	213	66.4 ± 3.2	57.2 (6.9–258.3)	211	61.6 ± 3.1	49.3 (4.5–270.5)	1655	84.8 ± 1.5	71.8 (5.0–802.7)	206	106.6 ± 4.8	94.6 (14.5–478.8)
*Plausible energy reporters*	543	100.7 ± 3.3 *	84.8 (11.0- 802.7)	120	72.5 ± 4.3 *	63.0 (13.3–258.3)	76	74.8 ± 5.6 *	61.1 (8.8–244.5)	433	103.0 ± 3.8 *	87.2 (5.9–802.7)	45	142.0 ± 12.9 *	127.0 (28.6–478.8)
* Men*	232	102.9 ± 5.3 ^§^	87.1 (14.1–802.7)	68	74.2 ± 5.4	64.7 (13.3–210.6)	48	77.7 ± 7.7	57.2 (20.6–244.5)	158	112.5 ± 7.1	95 (14.1–802.7)	24	144.1 ± 16.1	126.7 (28.6–315.3)
* Women*	311	99.0 ± 4.2 ^†^	84.0 (11.0–788.6)	52	70.2 ± 6.8	57.8 (16.8–258.3)	28	69.8 ± 7.5	64.3 (8.8–181.7)	275	97.5 ± 4.3	84 (5.9–788.6)	21	139.7 ± 21.2	131.3 (34.9–478.8)
*Non-Plausible energy reporters*	1466	78.4 ± 1.4	66.2 (5.0–410.6)	93	58.6 ± 4.6	48.7 (6.9–255.0)	135	54.2 ± 3.6	41.3 (4.5–270.5)	1222	78.4 ± 1.5	66.9 (5.0–408.3)	161	96.8 ± 4.6	86.9 (14.5–410.6)
* Men*	781	77.4 ± 2.0	63.9 (5.0–410.6)	58	54.5 ± 4.7	45.9 (6.9–172.8)	89	54.5 ± 4.6	40.9 (4.5–270.5)	640	78.5 ± 2.2	66 (5.0–408.3)	75	98.2 ± 7.7	83.9 (16.2–410.6)
* Women*	685	79.6 ± 1.9	68.3 (8.0–289.5)	35	65.5 ± 9.3	60.7 (11.8–255.0)	46	53.8 ± 6.0	42.3 (9.9–234.3)	582	78.3 ± 2.1	67.3 (8.0–343.4)	86	95.4 ± 5.5	87 (14.5–289.5)

Results are expressed as the mean ± standard error of the mean and median with range (in brackets). * *t*-test or Mann-Whitney U test: significant differences between plausible and non-plausible energy reporters in the whole population, (*p* < 0.05); ^§^ significant differences between plausible and non-plausible energy reporters men in the whole population (*p* < 0.05); ^†^ significant differences between plausible and non-plausible energy reporters women in the whole population (*p* < 0.05); there were significant differences between plausible and non-plausible energy reporters within sexes into each age group (*p* < 0.05).

**Table 4:** Percentage of the population with inadequate intake of zinc, selenium and vitamins A, for the whole population and for the plausible energy reporters by age.

	Total		Children		Adolescents		Adults		Elderly	
			9–12 Years		13–17 Years		18–64 Years		65–75 Years	
	Spain	EFSA	Spain	EFSA	Spain	EFSA	Spain	EFSA	Spain	EFSA
**Zinc (%)**										
*Whole population*	92	83	82	31	89	65	92	86	96	92
* Men*	86	69	80	30	85	59	86	72	93	84
* Women*	97	96	85	33	95	77	97	99	99	100
*Plausible energy reporters*	80	65	75	15	75	38	81	73	84	78
* Men*	64	31	74	15	69	27	59	31	75	58
* Women*	93	90	77	15	86	57	93	97	95	100
**Selenium (%)**										
*Whole population*	15	25	2	4	4	16	16	26	22	32
* Men*	16	18	2	5	3	12	18	19	22	22
* Women*	14	32	3	3	7	24	14	33	21	41
*Plausible energy reporters*	4	9	0	0	0	3	4	11	4	7
* Men*	3	3	0	0	0	0	3	3	4	4
* Women*	5	14	0	0	0	7	5	16	5	10
**Vitamin A (%)**										
*Whole population*	74	60	57	36	78	64	74	61	75	60
* Men*	78	64	57	33	80	66	80	57	80	65
* Women*	69	56	57	41	73	62	69	66	70	56
*Plausible energy reporters*	58	39	51	23	68	46	59	42	47	24
* Men*	63	40	53	18	71	46	66	44	63	33
* Women*	54	38	48	31	64	46	56	40	29	14
**Vitamin E (%)**										
*Whole population*	80	80	62	66	72	76	80	79	90	91
* Men*	78	82	63	69	72	79	78	82	89	92
* Women*	82	77	60	62	70	70	82	76	92	90
*Plausible energy reporters*	62	59	51	57	54	61	62	58	76	76
* Men*	56	61	53	59	54	65	52	58	79	83
* Women*	67	59	48	54	54	54	67	57	71	67
**Vitamin C (%)**										
*Whole population*	29	56	41	37	47	67	29	58	15	42
* Men*	32	60	39	37	48	69	31	62	19	44
* Women*	27	52	45	38	46	64	27	53	11	40
*Plausible energy reporters*	20	42	36	29	36	55	19	45	7	20
* Men*	21	42	29	26	35	56	19	44	8	17
* Women*	20	42	44	33	36	54	19	45	5	24

Results are expressed in percentage. Recommended daily intakes for Spain [20] and Europe [21]. Adequacy was calculated comparing with 80% of the Spanish DRV and EFSA PRI or AI.

## Supplementary Materials

The following are available online at www.mdpi.com/2072-6643/9/7/697/s1, Table S1: Daily zinc intake and distribution by sex and age group in the ANIBES Study population, Table S2: Daily selenium intake and distribution by sex and age group in the ANIBES Study population, Table S3: Daily vitamin A intake and distribution by sex and age group in the ANIBES Study population, Table S4: Daily retinol intake and distribution by sex and age group in the ANIBES Study population, Table S5: Daily carotenes intake and distribution by sex and age group in the ANIBES Study population, Table S6: Daily vitamin E intake and distribution by sex and age group in the ANIBES Study population, Table S7: Daily vitamin C intake and distribution by sex and age group in the ANIBES Study population, Table S8: Dietary sources of zinc (%) from food groups/subgroups by sex and age groups in the ANIBES Spanish population; Table S9: Dietary sources of selenium (%) from food groups/subgroups by sex and age groups in the ANIBES Spanish population, Table S10: Dietary sources of vitamin A (%) from food groups/subgroups by sex and age groups in the ANIBES Spanish population, Table S11: Dietary sources of retinol (%) from food groups/subgroups by sex and age groups in the ANIBES Spanish population, Table S12: Dietary sources of carotenes (%) from food groups/subgroups by sex and age groups in the ANIBES Spanish population, Table S13: Dietary sources of vitamin E (%) from food groups/subgroups by sex and age groups in the ANIBES Spanish population, Table S14: Dietary sources of vitamin C (%) from food groups/subgroups by sex and age groups in the ANIBES Spanish population.

## References

[1] World Health Organization (WHO) (2014). Global Status Report on Noncommunicables Diseases. http://apps.who.int/iris/bitstream/10665/148114/1/9789241564854_eng.pdf?ua=1.

[2] Ezzati M., Riboli E. (2013). Behavioral and dietary risk factors for noncommunicable diseases. N. Engl. J. Med..

[3] Ríos-Hoyo A., Cortés M.J., Ríos-Ontiveros H., Meaney E., Ceballos G., Gutiérrez-Salmeán G. (2014). Obesity, metabolic syndrome, and dietary therapeutical approaches with a special focus on nutraceuticals (polyphenols): A Mini-Review. Int. J. Vitam. Nutr. Res..

[4] Elmadfa I., Meyer A.L. (2010). Importance of food composition data to nutrition and public health. Eur. J. Clin. Nutr..

[5] King J.C., Brown K.H., Gibson R.S., Krebs N.F., Lowe N.M., Siekmann J.H., Raiten D.J. (2016). Biomarkers of nutrition for development (bond)-zinc review. J. Nutr..

[6] King J.C., Cousins R., Ross A.C., Caballero B., Cousins R.J., Tucker K.L., Ziegler T.R. (2014). Zinc. Modern Nutrition in Health and Disease.

[7] Burk R.F., Hill K.E. (2015). Regulation of selenium metabolism and transport. Annu. Rev. Nutr..

[8] EFSA Panel on Dietetic Products, Nutrition and Allergies (NDA) (2015). Scientific Opinion on Dietary Reference Values for vitamin A. EFSA J..

[9] Álvarez R., Vaz B., Gronemeyer H., de Lera Á.R. (2014). Functions, therapeutic applications, and synthesis of retinoids and carotenoids. Chem. Rev..

[10] Albahrani A.A., Greaves R.F. (2016). Fat-soluble vitamins: clinical indications and current challenges for chromatographic measurement. Clin. Biochem. Rev..

[11] European Food Safety Authority (EFSA) (2013). Scientific Opinion on Dietary Reference Values for vitamin C. EFSA Panel on Dietetic Products, Nutrition and Allergies (NDA). EFSA J..

[12] Archer E., Hand GA., Blair SN. (2013). Validity of U.S. nutritional surveillance: National Health and Nutrition Examination Survey caloric energy intake data, 1971–2010. PLoS ONE.

[13] Varela Moreiras G., Ávila J.M., Ruiz E. (2015). Energy balance, a new paradigm and methodological issues: The ANIBES study in Spain. Nutr. Hosp..

[14] Ruiz E., Ávila J.M., del Pozo S., Rodriguez P., Aranceta Bartrina J., Gil A., González-Gross M., Ortega R.M., Serra-Majem L., Varela-Moreiras G. (2016). Macronutrient distribution and dietary sources in the spanish population: Findings from the ANIBES Study. Nutrients.

[15] Olza J., Aranceta Bartrina J., González-Gross M., Ortega R.M., Serra-Majem L., Varela-Moreiras G., Gil A. (2017). Reported dietary intake, disparity between the reported consumption and the level needed for adequacy and food sources of calcium, phosphorus, magnesium and vitamin D in the Spanish population: Findings from the ANIBES study. Nutrients.

[16] Samaniego-Vaesken M.L., Partearroyo T., Olza J., Aranceta-Bartrina J., Gil A., González-Gross M., Ortega R.M., Serra-Majem L., Varela-Moreiras G. (2017). Iron intake and dietary sources in the spanish population: Findings from the ANIBES Study. Nutrients.

[17] Ruiz E., Ávila J.M., Castillo A., Valero T., del Pozo S., Rodriguez P., Aranceta-Bartrina J., Gil A., González-Gross M., Ortega R.M. (2015). The ANIBES study on energy balance in Spain: Design, Protocol and Methodology. Nutrients.

[18] Nissensohn M., Sánchez-Villegas A., Ortega R.M., Aranceta-Bartrina J., Gil Á., González-Gross M., Varela-Moreiras G., Serra-Majem L. (2016). Beverage consumption habits and association with total water and energy intakes in the spanish population: Findings of the ANIBES Study. Nutrients.

[19] Moreiras O., Carbajal A., Cabrera L., Cuadrado C. (2013). Tablas de Composición de Alimentos/Guía de Prácticas.

[20] Carbajal Á, García-Arias M.T., García-Fernández M.C. (2003). Ingestas Recomendadas de Energía y Nutrientes. Nutrición y Dietética.

[21] European Food Safety Authority (EFSA) Dietary Reference Values and Dietary Guidelines. https://www.efsa.europa.eu/en/topics/topic/drv.

[22] European Food Safety Authority (EFSA) Example of a Protocol for Identification of Misreporting (Under- and Over-Reporting of Energy Intake) Based on the PILOT-PANEU Project. http://www.efsa.europa.eu/sites/default/files/efsa_rep/blobserver_assets/3944A-8-2-1.pdf.

[23] Schofield W.N. (1985). Predicting basal metabolic rate, new standards and review of previous work. Hum. Nutr. Clin. Nutr..

[24] Mielgo-Ayuso J., Aparicio-Ugarriza R., Castillo A., Ruiz E., Ávila J.M., Aranceta Bartrina J., Gil A., Ortega R.M., Serra-Majem L., Varela-Moreiras G. (2016). Physical Activity patterns of the spanish population are mostly determined by sex and age: Findings in the ANIBES Study. PLoS ONE.

[25] Jansen E., Ruskovska T. (2015). Serum biomarkers of (anti) oxidant status for epidemiological studies. Int. J. Mol. Sci..

[26] Archer E., Pavela G., Lavie C.J. (2015). The inadmissibility of what we eat in America and NHANES dietary data in nutrition and obesity research and the scientific formulation of national dietary guidelines. Mayo Clin. Proc..

[27] Archer E., Pavela G., Lavie C.J. (2015). A discussion of the refutation of memory-based dietary assessment methods (m-bms): The rhetorical defense of pseudoscientific and inadmissible evidence. Mayo Clin. Proc..

[28] European Food Safety Authority (EFSA) (2014). Guidance on the EU Menu methodology. EFSA J..

[29] Varela-Moreiras G., Ruiz E., Valero T., Avila J.M., del Pozo S. (2013). The Spanish diet: An update. Nutr. Hosp..

[30] López-Sobaler A.M., Aparicio A., González-Rodríguez L.G., Cuadrado-Soto E., Rubio J., Marcos V., Sanchidrián R., Santos S., Pérez-Farinós N., Dal Re M.Á. (2017). Adequacy of usual vitamin and mineral intake in spanish children and adolescents: ENALIA study. Nutrients.

[31] Agencia Española de Seguridad Alimentaria y Nutrición (AESAN) Encuesta Nacional de Ingesta Dietética Española 2011. http://www.laboratoriolcn.com/f/docs/Valoracion_nutricional_ENIDE_micronutrientes.pdf.

[32] Mensink G.B., Fletcher R., Gurinovic M., Huybrechts I., Lafay L., Serra-Majem L., Szponar L., Tetens I., Verkaik-Kloosterman J., Baka A. (2013). Mapping low intake of micronutrients across Europe. Br. J. Nutr..

[33] Max Rubner-Institut (2008). Nationale Verzehrsstudie II. Ergebnisbericht Teil 2. Die bundesweite Befragung zur Ernährung von Jugendlichen und Erwachsenen.

[34] Lyhne N., Christensen T., Groth M.V., Fagt S., Biltoft-Jensen A., Hartkopp H., Hinsch H.J., Matthiessen J., Møller A., Saxholt E. (2005). Danskernes Kostvaner 2000–2002—Hovedresultater [Dietary Habits of Denmark 2000–2002].

[35] Paturi M., Tapanainen H., Reinivuo H., Pietinen P. (2008). The National FINDIET 2007 Survey.

[36] Peltonen M., Harald K., Männistö S., Saarikoski L., Peltomäki P., Lund L., Sundvall J., Juolevi A., Laatikainen T., Aldén-Nieminen H. (2008). The National FINRISK 2007 Study.

[37] Turrini A., Saba A., Perrone D., Cialfa E., D’Amicis A. (2001). Food consumption patterns in Italy: The INNCA Study 1994–1996. Eur. J. Clin. Nutr..

[38] Becker W., Pearson M. (2002). Riksmaten 1997–1998. Dietary Habits and Nutrient Intake in Sweden 1997–1998 (In Swedish/English Summary).

[39] Harrington J., Perry I., Lutomski J., Morgan K., McGee H., Shelley E., Watson D., Barry M. (2008). SLÁN 2007: Survey of Lifestyle, Attitudes and Nutrition in Ireland; Dietary Habits of the Irish Population, Department of Health and Children.

[40] Hulshof K.F.A.M., Ocke M.C., van Rossum C.T.M., Buurma-Rethans E.J.M., Brants H.A.M., Drijvers J.J.M.M., ter Doest D. (2004). Resultaten van de Voedselconsumptiepeiling 2003. Results of the National Food Consumption Survey 2003.

[41] Henderson L., Irving K., Gregory J., Bates C.J., Prentice A., Swan G., Farron M. (2003). Vitamin and Mineral Intake and Urinary Analyses. The National Diet and Nutrition Survey: Adults Aged 19 to 64 Year.

[42] Serra-Majem L., Ribas L., Pérez-Rodrigo C., García-Closas R., Peña-Quintana L., Aranceta J. (2002). Determinants of nutrient intake among children and adolescents: results from the enKid study. Ann. Nutr. Metab..

[43] Mensink G.B., Beitz R. (2004). Food and nutrient intake in East and West Germany, 8 years after the reunification —The German Nutrition Survey 1998. Eur. J. Clin. Nutr..

[44] Szponar L., Sekuła W., Rychlik E., Ołtarzewski M., Figurska K. (2003). Badania Indywidualnego Spozycia Zywnosci i Stanu Odzywienia w Gospodarstwach Domowych (Household Food Consumption and Anthropometric Survey).

[45] Dubuisson C., Lioret S., Touvier M., Dufour A., Calamassi-Tran G., Volatier J.L., Lafay L. (2010). Trends in food and nutritional intakes of French adults from 1999 to 2007: Results from the INCA surveys. Br. J. Nutr..

[46] Agence Française de Sécurité Sanitaire des Aliments (AFSSA) (2009). Étude Individuelle Nationale des Consommations Alimentaires 2 (INCA 2) (2006–2007).

[47] Nicholson S.K., Roberts C., Prynne C.J., Pot G.K., Olson A., Fitt E., Cole D., Teucher B., Bates B., Henderson H. (2011). National diet and nutrition survey: UK food consumption and nutrient intakes from the first year of the rolling programme and comparisons with previous surveys. Br. J. Nutr..

[48] Food D.T.U. (2010). Danskernes Kostvaner 2003–2008 (Dietary Habits in Denmark 2003–2008).

[49] Elmadfa I. (2009). European Nutrition and Health Report 2009.

[50] Kjøller M., Juel K., Kamper-Jørgensen F. (2007). Folkesundhedsrapporten, Danmark 2007 [The Report on Public Health, Denmark 2007].

[51] Mendez M.A., Popkin B.M., Buckland G., Schroder H., Amiano P., Barricarte A., Huerta J.M., Quirós J.R., Sánchez M.J., González C.A. (2011). Alternative methods of accounting for underreporting and overreporting when measuring dietary intake-obesity relations. Am. J. Epidemiol..

[52] Poslusna K., Ruprich J., de Vries J.H., Jakubikova M., van’t Veer P. (2009). Misreporting of energy and micronutrient intake estimated by food records and 24 h recalls, control and adjustment methods in practice. Br. J. Nutr..

