# Supplementary material for: Reported Dietary Intake and Food Sources of Zinc, Selenium, and Vitamins A, E and C in the Spanish Population: Findings from the ANIBES Study
Source: Nutrients. 2017 Jul 6;9(7):697. doi: 10.3390/nu9070697 (PMC5537812; doi:10.3390/nu9070697)
Supplement: Supplementary file 1 [file nutrients-09-00697-s001.zip › nutrients-200193-supplementary.pdf]

Table S1. Daily zinc intake and distribution by sex and age group in the ANIBES Study population.

ZINC (mg/d)	*n*	Mean	SD	SEM	P5	P25	P50 Median	P75	P95	Minimum	Maximum
**Total**	**2009**	**8.1**	**2.6**	**0.1**	**4.6**	**6.3**	**7.7**	**9.5**	**12.9**	**2.3**	**27.3**
Men	1013	8.8	2.9	0.1	4.9	6.8	8.4	10.4	13.9	2.3	27.3
Women	996	7.4	2.2	0.1	4.4	5.9	7.2	8.5	11.2	2.9	19.5
**Children 9–12 years**	**213**	**8.3**	**2.1**	**0.1**	**3.7**	**17.3**	**8.2**	**4.8**	**6.7**	**9.6**	**12.2**
Men	126	8.6	2.2	0.2	3.7	17.3	8.2	5.3	7.3	9.9	12.6
Women	87	7.8	2.0	0.2	4.3	12.5	7.6	4.7	6.2	9.3	11.4
**Adolescents 13–17 years**	**211**	**8.6**	**2.6**	**0.2**	**2.9**	**18.6**	**8.3**	**4.9**	**6.8**	**10.0**	**13.2**
Men	137	9.2	2.6	0.2	2.9	18.6	8.7	5.5	7.5	10.9	13.8
Women	74	7.4	2.3	0.3	3.6	13.5	7.5	3.7	5.8	8.8	12.8
**Adults 18–64 years**	**1655**	**8.2**	**2.7**	**0.1**	**2.3**	**27.3**	**7.7**	**4.7**	**6.3**	**9.7**	**13.0**
Men	798	8.9	2.9	0.1	2.3	27.3	8.6	4.9	6.8	10.5	14.4
Women	857	7.5	2.2	0.1	2.9	19.5	7.2	4.4	5.9	8.6	11.4
**Elderly 65–75 years**	**206**	**7.4**	**2.4**	**0.2**	**3.1**	**20.0**	**7.1**	**4.3**	**5.7**	**8.5**	**11.2**
Men	99	8.1	2.7	0.3	3.7	20.0	7.5	4.8	6.2	9.5	13.4
Women	107	6.8	1.9	0.2	3.1	12.3	6.8	3.9	5.4	7.8	10.5

Table S2. Daily selenium intake and distribution by sex and age group in the ANIBES Study population.

SELENIUM (mg/d)	*n*	Mean	SD	SEM	P5	P25	P50 Median	P75	P95	Minimum	Maximum
**Total**	**2009**	75	28	1	36	55	72	91	127	14	265
Men	1013	81	29	1	42	61	77	100	136	20	188
Women	996	69	26	1	33	51	67	84	112	14	265
**Children 9–12 years**	**213**	**77**	**25**	**2**	**37**	**59**	**76**	**92**	**122**	**9**	**180**
Men	126	79	27	2	39	59	76	96	128	9	180
Women	87	74	23	2	37	57	75	91	107	24	140
**Adolescents 13–17 years**	**211**	**80**	**29**	**2**	**38**	**58**	**77**	**98**	**135**	**26**	**164**
Men	137	85	29	2	40	64	81	107	136	26	164
Women	74	71	27	3	34	52	67	86	131	30	150
**Adults 18–64 years**	**1655**	**76**	**28**	**1**	**36**	**55**	**72**	**92**	**127**	**14**	**265**
Men	798	82	29	1	42	62	79	101	137	20	198
Women	857	70	26	1	33	51	67	85	114	14	265
**Elderly 65–75 years**	**206**	**70**	**28**	**2**	**34**	**51**	**65**	**83**	**117**	**23**	**221**
Men	99	75	30	3	41	58	70	86	124	28	221
Women	107	64	24	2	31	46	61	80	109	23	144

Table S3. Daily vitamin A intake and distribution by sex and age group in the ANIBES Study population.

VITAMIN A (µg RE/d)	*n*	Mean	SD	SEM	P5	P25	P50 Median	P75	P95	Minimum	Maximum
**Total**	**2009**	**668**	**850**	**19**	**158**	**318**	**477**	**707**	**1646**	**2**	**11017**
Men	1013	691	934	29	171	320	478	707	1731	38	11017
Women	996	644	754	24	151	315	474	705	1509	2	7505
**Children 9–12 years**	**213**	**664**	**632**	**43**	**164**	**326**	**496**	**759**	**1914**	**79**	**5991**
Men	126	702	577	51	164	326	531	883	1965	79	3196
Women	87	609	703	75	164	305	427	656	1079	120	5991
**Adolescents 13–17 years**	**211**	**570**	**475**	**33**	**165**	**309**	**426**	**687**	**1446**	**108**	**3434**
Men	137	582	488	42	165	319	446	718	1449	109	3434
Women	74	546	452	53	164	298	383	651	1433	108	2831
**Adults 18–64 years**	**1655**	**672**	**866**	**21**	**159**	**320**	**479**	**710**	**1605**	**2**	**11017**
Men	798	697	958	34	173	324	484	707	1663	38	11017
Women	857	650	770	26	147	315	474	714	1593	2	7505
**Elderly 65–75 years**	**206**	**658**	**872**	**61**	**156**	**313**	**489**	**683**	**1503**	**78**	**7796**
Men	99	708	1032	104	182	322	475	699	1734	96	7796
Women	107	612	695	67	153	281	492	678	1348	78	6584

Table S4. Daily retinol intake and distribution by sex and age group in the ANIBES Study population.

RETINOL (µg/d)	*n*	Mean	SD	SEM	P5	P25	P50 Median	P75	P95	Minimum	Maximum
**Total**	**2009**	**364**	**813**	**18**	**48**	**117**	**187**	**293**	**1118**	**0**	**10881**
Men	996	399	899	28	52	125	199	316	1381	0	10881
Women	1013	327	714	23	41	109	176	275	956	0	7440
**Children 9–12 years**	**213**	**420**	**619**	**42**	**72**	**154**	**227**	**371**	**1569**	**18**	**5950**
Men	126	461	556	50	72	163	262	413	1895	18	2802
Women	87	362	701	75	77	137	211	324	908	46	5950
**Adolescents 13–17 years**	**211**	**343**	**419**	**29**	**66**	**141**	**218**	**343**	**1256**	**0**	**2697**
Men	137	359	433	37	79	156	237	361	1342	0	2697
Women	74	312	391	45	54	124	204	319	1208	21	2392
**Adults 18–64 years**	**1655**	**363**	**830**	**20**	**48**	**119**	**186**	**292**	**1096**	**0**	**10881**
Men	798	395	926	33	52	126	197	315	1290	0	10881
Women	857	333	729	25	41	111	177	275	969	0	7440
**Elderly 65–75 years**	**206**	**309**	**817**	**57**	**37**	**96**	**163**	**260**	**633**	**3**	**7407**
Men	99	361	977	98	32	105	167	260	673	3	7407
Women	107	261	634	61	38	86	160	262	543	9	6494

Table S5. Daily carotenes intake and distribution by sex and age group in the ANIBES Study population.

CAROTENES (µg/d)	*n*	Mean	SD	SEM	P5	P25	P50 Median	P75	P95	Minimum	Maximum
**Total**	**2009**	**1735**	**1551**	**35**	**242**	**688**	**1342**	**2249**	**4655**	**13**	**13962**
Men	996	1652	1474	46	221	658	1231	2171	4599	14	10960
Women	1013	1820	1622	51	264	724	1415	2330	4714	13	13962
**Children 9–12 years**	**213**	**1331**	**1137**	**78**	**192**	**500**	**995**	**1831**	**3681**	**42**	**6222**
Men	126	1283	1147	102	178	444	980	1799	3480	50	5754
Women	87	1402	1125	121	244	625	1023	1930	3681	42	6222
**Adolescents 13–17 years**	**211**	**1254**	**1152**	**79**	**141**	**483**	**882**	**1598**	**3695**	**45**	**6805**
Men	137	1227	1165	100	147	476	873	1441	3827	79	6197
Women	74	1303	1136	132	103	621	993	1673	3695	45	6805
**Adults 18–64 years**	**1655**	**1760**	**1577**	**39**	**245**	**699**	**1355**	**2257**	**4714**	**13**	**13962**
Men	798	1696	1513	54	243	681	1313	2198	4698	14	10960
Women	857	1819	1633	56	245	728	1419	2291	4731	13	13962
**Elderly 65–75 years**	**206**	**2082**	**1750**	**122**	**292**	**872**	**1618**	**2659**	**5095**	**97**	**11643**
Men	99	2068	1506	151	290	1022	1705	2703	5304	123	6851
Women	107	2095	1956	189	292	804	1528	2659	5095	97	11643

Table S6. Daily vitamin E intake and distribution by sex and age group in the ANIBES Study population.

VITAMIN E (mg α-TE/d)	*n*	Mean	SD	SEM	P5	P25	P50 Median	P75	P95	Minimum	Maximum
**Total**	**2009**	**7.0**	**3.8**	**0.1**	**2.4**	**4.5**	**6.3**	**8.8**	**14.0**	**0.7**	**55.2**
Men	996	7.3	4.2	0.1	2.3	4.7	6.5	9.1	14.8	0.7	55.2
Women	1013	6.7	3.4	0.1	2.4	4.3	6.1	8.5	13.1	0.7	27.5
**Children 9–12 years**	**213**	**7.4**	**4.0**	**0.3**	**2.6**	**4.5**	**6.3**	**9.5**	**15.5**	**0.7**	**27.6**
Men	126	7.4	4.0	0.4	2.6	4.8	6.1	9.4	15.3	0.7	27.6
Women	87	7.5	4.0	0.4	2.6	4.2	6.6	9.7	16.0	2.2	19.1
**Adolescents 13–17 years**	**211**	**7.5**	**4.5**	**0.3**	**2.4**	**4.4**	**6.4**	**9.7**	**15.7**	**1.1**	**31.0**
Men	137	7.6	4.2	0.4	2.4	4.6	6.5	9.6	15.8	1.1	24.0
Women	74	7.4	5.0	0.6	2.4	4.1	6.3	10.0	14.9	1.7	31.0
**Adults 18–64 years**	**1655**	**7.1**	**3.9**	**0.1**	**2.4**	**4.6**	**6.5**	**8.9**	**14.2**	**0.7**	**55.2**
Men	798	7.4	4.3	0.2	2.4	4.7	6.7	9.1	14.8	0.9	55.2
Women	857	6.8	3.4	0.1	2.4	4.4	6.3	8.7	13.4	0.7	27.5
**Elderly 65–75 years**	**206**	**5.9**	**2.9**	**0.2**	**2.2**	**3.9**	**5.2**	**7.8**	**11.0**	**1.7**	**16.6**
Men	99	6.3	3.0	0.3	2.3	3.9	5.8	8.1	12.1	1.8	16.6
Women	107	5.6	2.7	0.3	2.1	3.9	4.9	6.7	10.7	1.7	15.7

Table S7. Daily vitamin C intake and distribution by sex and age group in the ANIBES Study population.

VITAMIN C (mg/d)	*n*	Mean	SD	SEM	P5	P25	P50 Median	P75	P95	Minimum	Maximum
**Total**	**2009**	**84.4**	**61.5**	**1.4**	**20.4**	**42.6**	**71.3**	**109.6**	**193.3**	**5.0**	**802.7**
Men	996	83.2	63.9	2.0	19.0	39.7	68.9	106.6	199.3	5.0	802.7
Women	1013	85.6	58.8	1.9	21.7	46.2	72.8	112.9	188.7	8.0	788.6
**Children 9–12 years**	**213**	**66.4**	**46.1**	**3.2**	**16.8**	**34.4**	**57.2**	**86.3**	**156.8**	**6.9**	**258.3**
Men	126	65.1	42.1	3.7	17.0	35.5	56.6	82.8	145.4	6.9	210.6
Women	87	68.3	51.5	5.5	16.8	32.1	58.5	95.1	156.8	11.8	258.3
**Adolescents 13–17 years**	**211**	**61.6**	**45.5**	**3.1**	**15.2**	**30.3**	**49.3**	**77.1**	**164.0**	**4.5**	**270.5**
Men	137	62.6	48.0	4.1	14.6	29.4	48.3	77.1	172.2	4.5	270.5
Women	74	59.9	40.7	4.7	15.6	32.3	50.6	74.6	151.7	8.8	234.3
**Adults 18–64 years**	**1655**	**84.8**	**61.8**	**1.5**	**19.5**	**43.3**	**71.8**	**110.2**	**193.8**	**5.0**	**802.7**
Men	798	85.2	65.5	2.3	19.0	40.5	72.0	108.1	203.1	5.0	802.7
Women	857	84.5	58.2	2.0	19.5	45.8	71.8	111.8	188.6	5.9	788.6
**Elderly 65–75 years**	**206**	**106.6**	**68.3**	**4.8**	**28.1**	**59.1**	**94.6**	**134.0**	**234.1**	**14.5**	**478.8**
Men	99	109.4	72.3	7.3	23.8	56.6	96.6	134.0	250.9	16.2	410.6
Women	107	104.1	64.6	6.2	29.0	59.1	91.6	136.0	204.1	14.5	478.8

Table S8. Dietary sources of zinc (%) from food groups/subgroups by sex and age groups in the ANIBES Spanish population.

ZINC	Total 9–75	Children 9–12	Adolescents 13–17	Adults 18–64	Elderly 65–75
(%)	2009	213	211	1655	206
**Alcoholic beverages**	**0.59**	**-**	**0.01**	**0.64**	**0.88**
High alcohol content beverages	-	-	-	-	-
Low alcohol content beverages	0.59	-	0.01	0.64	0.88
**Appetizers**	**0.30**	**0.65**	**0.49**	**0.29**	**0.07**
**Cereals/Grains**	**25.46**	**25.04**	**26.32**	**25.42**	**25.90**
Grains and flours	1.96	1.59	1.97	2.01	1.75
Breakfast cereals and cereal bars	0.17	0.04	0.09	0.20	0.15
Bread	16.93	15.31	15.79	16.90	18.81
White bread	14.38	14.32	14.92	14.17	15.85
Brown bread	2.19	0.80	0.64	2.35	2.67
Others breads	0.36	0.19	0.22	0.39	0.29
Pasta	3.22	3.81	4.23	3.25	2.01
Bakery and pastry	3.19	4.30	4.25	3.06	3.17
**Eggs**	**4.56**	**4.29**	**4.50**	**4.52**	**5.48**
**Fish**	**5.72**	**3.69**	**3.53**	**5.80**	**6.90**
White fish	1.11	1.03	0.62	1.04	2.05

ZINC	Total 9–75	Children 9–12	Adolescents 13–17	Adults 18–64	Elderly 65–75
**(%)**	**2009**	**213**	**211**	**1655**	**206**
Oily fish	0.90	0.48	0.52	0.91	1.07
Shellfish	2.37	1.69	1.54	2.44	2.54
Canned fish	1.34	0.49	0.85	1.42	1.25
**Fruits**	**3.52**	**2.46**	**1.88**	**3.43**	**5.88**
**Meat and meat products**	**28.53**	**29.37**	**29.83**	**28.74**	**24.72**
Meat	19.17	18.30	19.03	19.42	16.97
Red meat	8.63	9.01	7.69	8.63	8.57
White meat	5.45	4.57	5.64	5.64	4.26
Poultry	5.09	4.71	5.70	5.15	4.14
Viscera and offal	0.46	0.11	0.07	0.48	0.63
Sausages and other meat products	8.90	10.97	10.73	8.84	7.12
**Milk and dairy products**	**15.82**	**18.88**	**16.70**	**15.59**	**15.80**
Milk	6.67	8.66	7.31	6.39	7.64
Whole milk	2.01	4.10	3.39	1.83	2.09
Semi skimmed milk	2.99	3.82	2.86	2.91	2.90
Skimmed milk	1.53	0.72	1.03	1.51	2.50
Other dairy	0.13	0.01	0.04	0.14	0.15

ZINC (%)	Total 9–75 2009	Children 9–12 213	Adolescents 13–17 211	Adults 18–64 1655	Elderly 65–75 206
Yogurt and fermented milk	2.76	3.18	2.01	2.65	3.69
Skimmed Fermented milk	0.22	0.58	0.17	0.19	0.41
Whole fermented milk	0.14	0.04	0.03	0.14	0.15
Skimmed milk yogurts	0.71	0.12	0.15	0.71	1.05
Whole milk yogurt	1.70	2.45	1.66	1.61	2.08
Cheese	5.75	5.07	6.05	5.96	4.23
Other dairy products	0.64	1.97	1.32	0.59	0.23
**Non-alcoholic beverages**	**0.87**	**0.89**	**0.83**	**0.89**	**0.93**
Water	-	-	-	-	-
Coffee and herbal teas	0.04	-	-	0.04	0.07
Sugared soft drinks	-	-	-	-	-
Unsweetened soft drinks	-	-	-	-	-
Sports drinks	-	-	-	-	-
Juices and nectars	0.52	0.86	0.67	0.50	0.47
Energy drinks	-	-	-	-	-
Other non-alcoholic beverages	0.31	0.03	0.16	0.35	0.39
**Oils and fats**	**0.04**	**0.04**	**0.05**	**0.04**	**0.04**

ZINC	Total 9–75	Children 9–12	Adolescents 13–17	Adults 18–64	Elderly 65–75
**(%)**	**2009**	**213**	**211**	**1655**	**206**
Olive oil	-	-	-	-	-
Other oils	-	-	-	-	-
Butter, margarine and shortening	0.04	0.04	0.05	0.04	0.04
**Pulses**	**3.53**	**3.24**	**3.23**	**3.48**	**4.58**
**Sauces and condiments**	**0.49**	**0.56**	**0.53**	**0.50**	**0.35**
**Sugars and sweets**	**0.43**	**0.91**	**0.71**	**0.42**	**0.17**
Sugar	0.07	0.02	0.04	0.08	0.08
Chocolate	0.34	0.86	0.63	0.33	0.10
Jams and other	-	-	-	-	-
Other sweets	0.01	0.03	0.03	0.02	-
**Supplements and meal replacements**	**0.21**	**0.10**	**-**	**0.25**	**-**
**Ready-to-eat-meals**	**4.80**	**6.44**	**7.84**	**4.75**	**1.91**
**Vegetables**	**5.15**	**3.46**	**3.54**	**5.23**	**6.39**

Table S9. Dietary sources of selenium (%) from food groups/subgroups by sex and age groups in the ANIBES Spanish population.

SELENIUM	Total 9–75	Children 9–12	Adolescents 13–17	Adults 18–64	Elderly 65–75
(%)	2009	213	211	1655	206
**Alcoholic beverages**	**-**	**-**	**-**	**-**	**-**
High alcohol content beverages	-	-	-	-	-
Low alcohol content beverages	-	-	-	-	-
**Appetizers**	**0.16**	**0.22**	**0.20**	**0.17**	**0.06**
**Cereals/Grains**	**46.50**	**48.17**	**50.77**	**46.31**	**44.60**
Grains and flours	3.17	2.91	3.82	3.19	2.73
Breakfast cereals and cereal bars	0.12	0.01	0.08	0.14	0.12
Bread	29.66	28.47	29.30	29.63	30.97
White bread	25.77	26.83	27.91	25.47	26.60
Brown bread	3.13	1.25	1.00	3.35	3.73
Others breads	0.76	0.39	0.39	0.81	0.64
Pasta	10.15	12.81	13.78	10.04	7.23
Bakery and pastry	3.39	3.96	3.79	3.31	3.54
**Eggs**	**4.54**	**4.20**	**4.58**	**4.52**	**5.47**
**Fish**	**16.68**	**11.81**	**9.79**	**16.70**	**22.00**

SELENIUM	Total 9–75	Children 9–12	Adolescents 13–17	Adults 18–64	Elderly 65–75
**(%)**	**2009**	**213**	**211**	**1655**	**206**
White fish	5.21	5.71	3.33	4.85	9.19
Oily fish	3.58	2.24	1.85	3.57	4.58
Shellfish	4.03	2.27	2.33	4.20	4.44
Canned fish	3.85	1.60	2.28	4.08	3.78
**Fruits**	**1.42**	**0.95**	**0.85**	**1.43**	**2.22**
**Meat and meat products**	**14.93**	**15.42**	**15.89**	**15.20**	**11.91**
Meat	9.70	8.12	9.78	10.00	8.06
Red meat	1.25	0.87	0.97	1.28	1.21
White meat	4.06	3.27	3.99	4.23	3.21
Poultry	4.39	3.98	4.82	4.49	3.64
Viscera and offal	0.47	0.10	0.13	0.48	0.77
Sausages and other meat products	4.77	7.20	5.98	4.72	3.07
**Milk and dairy products**	**7.18**	**8.80**	**7.59**	**7.06**	**6.97**
Milk	2.76	3.59	3.11	2.65	3.17
Whole milk	0.83	1.82	1.40	0.76	0.83
Semi skimmed milk	1.25	1.46	1.30	1.21	1.23
Skimmed milk	0.63	0.30	0.39	0.62	1.05

SELENIUM (%)	Total 9–75	Children 9–12	Adolescents 13–17	Adults 18–64	Elderly 65–75
	**2009**	**213**	**211**	**1655**	**206**
Other dairy	0.05	0.01	0.02	0.05	0.05
Yogurt and fermented milk	0.98	1.04	0.71	0.94	1.43
Skimmed Fermented milk	0.07	0.22	0.06	0.06	0.18
Whole fermented milk	0.06	0.02	0.03	0.05	0.13
Skimmed milk yogurts	0.23	0.03	0.05	0.23	0.33
Whole milk yogurt	0.62	0.78	0.57	0.59	0.79
Cheese	2.80	2.54	2.79	2.89	1.93
Other dairy products	0.64	1.63	0.99	0.59	0.44
**Non-alcoholic beverages**	**0.70**	**0.77**	**0.71**	**0.75**	**0.47**
Water	-	-	-	-	-
Coffee and herbal teas	-	-	-	-	-
Sugared soft drinks	0.11	0.07	0.15	0.12	0.02
Unsweetened soft drinks	0.05	0.02	0.03	0.05	0.02
Sports drinks	-	-	-	-	-
Juices and nectars	0.36	0.67	0.48	0.35	0.19
Energy drinks	-	-	-	-	-
Other non-alcoholic beverages	0.19	0.02	0.05	0.23	0.24

SELENIUM	Total 9–75	Children 9–12	Adolescents 13–17	Adults 18–64	Elderly 65–75
(%)	2009	213	211	1655	206
**Oils and fats**	**-**	**-**	**-**	**-**	**-**
Olive oil	-	-	-	-	-
Other oils	-	-	-	-	-
Butter, margarine and shortening	-	-	-	-	-
**Pulses**	**1.58**	**1.44**	**1.37**	**1.55**	**2.10**
**Sauces and condiments**	**0.18**	**0.10**	**0.19**	**0.20**	**0.10**
**Sugars and sweets**	**0.93**	**2.22**	**2.25**	**0.84**	**0.27**
Sugar	0.01	-	-	0.01	0.01
Chocolate	0.91	2.21	2.24	0.82	0.25
Jams and other	0.01	-	-	0.01	0.02
Other sweets	-	-	-	-	-
**Supplements and meal replacements**	**0.13**	**0.08**	**-**	**0.16**	**-**
**Ready-to-eat-meals**	**2.62**	**4.09**	**4.16**	**2.57**	**1.07**
**Vegetables**	**2.43**	**1.74**	**1.65**	**2.56**	**2.77**

Table S10. Dietary sources of vitamin A (%) from food groups/subgroups by sex and age groups in the ANIBES Spanish population.

VITAMIN A	Total 9–75	Children 9–12	Adolescents 13–17	Adults 18–64	Elderly 65–75
(%)	2009	213	211	1655	206
**Alcoholic beverages**	**-**	**-**	**-**	**-**	**-**
High alcohol content beverages	-	-	-	-	-
Low alcohol content beverages	-	-	-	-	-
**Appetizers**	**0.18**	**0.26**	**0.11**	**0.17**	**0.18**
**Cereals/Grains**	**4.53**	**4.40**	**5.93**	**4.53**	**3.42**
Grains and flours	0.05	0.05	0.04	0.06	0.03
Breakfast cereals and cereal bars	-	-	-	-	-
Bread	-	-	-	-	-
White bread	-	-	-	-	-
Brown bread	-	-	-	-	-
Others breads	-	-	-	-	-
Pasta	0.05	0.05	0.04	0.07	-
Bakery and pastry	4.42	4.30	5.85	4.40	3.39
**Eggs**	**10.99**	**10.88**	**11.98**	**10.89**	**11.87**
**Fish**	**4.20**	**3.26**	**3.30**	**4.27**	**4.67**

VITAMIN A (%)	Total 9–75	Children 9–12	Adolescents 13–17	Adults 18–64	Elderly 65–75
	**2009**	**213**	**211**	**1655**	**206**
White fish	0.17	0.11	0.07	0.18	0.15
Oily fish	1.57	1.71	1.30	1.50	2.22
Shellfish	0.92	0.63	0.66	0.96	0.98
Canned fish	1.54	0.81	1.27	1.63	1.31
**Fruits**	**6.86**	**4.06**	**3.59**	**6.73**	**11.31**
**Meat and meat products**	**2.87**	**5.35**	**2.59**	**2.76**	**1.69**
Meat	0.02	0.01	-	-	0.04
Red meat	0.02	0.01	-	-	0.04
White meat	-	-	-	-	-
Poultry	-	-	-	-	-
Viscera and offal	0.63	0.15	-	0.68	0.63
Sausages and other meat products	2.23	5.18	2.59	2.06	1.02
**Milk and dairy products**	**21.73**	**30.31**	**29.72**	**21.56**	**16.41**
Milk	5.82	11.66	10.76	5.28	5.35
Whole milk	5.54	11.64	10.67	4.99	5.11
Semi skimmed milk	-	-	-	-	-
Skimmed milk	-	-	-	-	-

VITAMIN A (%)	Total 9–75	Children 9–12	Adolescents 13–17	Adults 18–64	Elderly 65–75
	**2009**	**213**	**211**	**1655**	**206**
Other dairy	0.28	0.02	0.09	0.30	0.24
Yogurt and fermented milk	2.47	3.02	2.59	2.34	3.22
Skimmed Fermented milk	0.04	0.11	0.04	0.03	0.08
Whole fermented milk	0.23	0.05	0.05	0.22	0.48
Skimmed milk yogurts	0.14	0.04	0.19	0.13	0.14
Whole milk yogurt	2.06	2.82	2.31	1.97	2.52
Cheese	10.31	9.82	12.42	10.78	6.31
Other dairy products	3.13	5.81	3.95	3.15	1.53
**Non-alcoholic beverages**	**1.24**	**2.37**	**2.18**	**1.18**	**0.83**
Water	-	-	-	-	-
Coffee and herbal teas	-	-	-	-	-
Sugared soft drinks	-	-	-	-	-
Unsweetened soft drinks	-	-	-	-	-
Sports drinks	-	-	-	-	-
Juices and nectars	1.24	2.37	2.18	1.18	0.83
Energy drinks	-	-	-	-	-
Other non-alcoholic beverages	-	-	-	-	-

VITAMIN A	Total 9–75	Children 9–12	Adolescents 13–17	Adults 18–64	Elderly 65–75
**(%)**	**2009**	**213**	**211**	**1655**	**206**
**Oils and fats**	**5.63**	**5.54**	**5.62**	**5.59**	**6.54**
Olive oil	0.38	0.34	0.25	0.39	0.47
Other oils	-	-	-	-	-
Butter, margarine and shortening	5.26	5.20	5.38	5.20	6.07
**Pulses**	**0.94**	**0.75**	**0.77**	**0.95**	**1.09**
**Sauces and condiments**	**3.60**	**3.70**	**4.75**	**3.72**	**1.65**
**Sugars and sweets**	**0.27**	**0.57**	**0.48**	**0.24**	**0.13**
Sugar	-	-	-	-	-
Chocolate	0.22	0.44	0.47	0.21	0.04
Jams and other	0.03	0.02	0.01	0.03	0.09
Other sweets	0.01	0.10	-	-	-
**Supplements and meal replacements**	**0.19**	**0.21**	**-**	**0.23**	**0.20**
**Ready-to-eat-meals**	**5.45**	**6.85**	**9.19**	**5.24**	**3.49**
**Vegetables**	**31.32**	**21.52**	**19.78**	**31.94**	**36.54**

Table S11. Dietary sources of retinol (%) from food groups/subgroups by sex and age groups in the ANIBES Spanish population.

RETINOL	Total 9–75	Children 9–12	Adolescents 13–17	Adults 18–64	Elderly 65–75
(%)	2009	213	211	1655	206
**Alcoholic beverages**	**-**	**-**	**-**	**-**	**-**
High alcohol content beverages	-	-	-	-	-
Low alcohol content beverages	-	-	-	-	-
**Appetizers**	**-**	**-**	**-**	**-**	**-**
**Cereals/Grains**	**8.27**	**7.34**	**9.67**	**8.45**	**6.09**
Grains and flours	-	-	-	-	-
Breakfast cereals and cereal bars	-	-	-	-	-
Bread	-	-	-	-	-
White bread	-	-	-	-	-
Brown bread	-	-	-	-	-
Others breads	-	-	-	-	-
Pasta	0.05	0.09	-	0.07	-
Bakery and pastry	8.21	7.24	9.67	8.37	6.09
**Eggs**	**22.58**	**18.84**	**20.35**	**22.49**	**27.16**
**Fish**	**11.42**	**6.53**	**6.93**	**11.68**	**14.77**
White fish	0.64	0.37	0.28	0.67	0.88

RETINOL	Total 9–75	Children 9–12	Adolescents 13–17	Adults 18–64	Elderly 65–75
**(%)**	**2009**	**213**	**211**	**1655**	**206**
Oily fish	3.28	2.54	1.88	3.18	4.84
Shellfish	2.62	1.59	1.79	2.75	3.37
Canned fish	4.89	2.02	2.98	5.08	5.68
**Fruits**	**-**	**-**	**-**	**-**	**-**
**Meat and meat products**	**2.80**	**5.47**	**2.59**	**2.69**	**1.60**
Meat	0.01	-	-	0.02	-
Red meat	-	-	-	-	-
White meat	-	-	-	-	-
Poultry	0.01	-	-	0.02	-
Viscera and offal	0.66	0.23	-	0.73	0.64
Sausages and other meat products	2.13	5.24	2.59	1.95	0.97
**Milk and dairy products**	**38.67**	**45.92**	**40.88**	**38.37**	**37.27**
Milk	10.03	16.86	14.98	9.39	9.76
Whole milk	9.46	16.79	14.87	8.77	9.24
Semi skimmed milk	-	-	-	-	-
Skimmed milk	-	-	-	-	-
Other dairy	0.57	0.07	0.11	0.62	0.52

RETINOL (%)	Total 9–75 2009	Children 9–12 213	Adolescents 13–17 211	Adults 18–64 1655	Elderly 65–75 206
Yogurt and fermented milk	7.42	6.62	5.41	7.06	11.90
Skimmed Fermented milk	0.32	0.55	0.40	0.24	0.87
Whole fermented milk	0.59	0.13	0.06	0.63	0.90
Skimmed milk yogurts	0.98	0.09	0.23	0.93	1.87
Whole milk yogurt	5.53	5.85	4.71	5.25	8.27
Cheese	16.38	13.57	15.53	17.08	12.57
Other dairy products	4.84	8.88	4.97	4.84	3.03
**Non-alcoholic beverages**	**-**	**-**	**-**	**-**	**-**
Water	-	-	-	-	-
Coffee and herbal teas	-	-	-	-	-
Sugared soft drinks	-	-	-	-	-
Unsweetened soft drinks	-	-	-	-	-
Sports drinks	-	-	-	-	-
Juices and nectars	-	-	-	-	-
Energy drinks	-	-	-	-	-
Other non-alcoholic beverages	-	-	-	-	-
**Oils and fats**	**8.81**	**7.95**	**8.03**	**8.87**	**9.86**

RETINOL	Total 9–75	Children 9–12	Adolescents 13–17	Adults 18–64	Elderly 65–75
**(%)**	**2009**	**213**	**211**	**1655**	**206**
Olive oil	-	-	-	-	-
Other oils	-	-	-	-	-
Butter, margarine and shortening	8.81	7.95	8.03	8.87	9.86
**Pulses**	**-**	**-**	**-**	**-**	**-**
**Sauces and condiments**	**1.98**	**0.86**	**2.20**	**2.12**	**0.84**
**Sugars and sweets**	**0.04**	**0.01**	**0.17**	**0.02**	**-**
Sugar	-	-	-	-	-
Chocolate	0.04	0.01	0.17	0.02	-
Jams and other	-	-	-	-	-
Other sweets	-	-	-	-	-
**Supplements and meal replacements**	**-**	**-**	**-**	**-**	**-**
**Ready-to-eat-meals**	**5.37**	**7.06**	**8.99**	**5.27**	**2.26**
**Vegetables**	**0.05**	**0.02**	**0.18**	**0.04**	**0.15**

Table S12. Dietary sources of carotenes (%) from food groups/subgroups by sex and age groups in the ANIBES Spanish population.

CAROTENES	Total 9–75	Children 9–12	Adolescents 13–17	Adults 18–64	Elderly 65–75
(%)	2009	213	211	1655	206
**Alcoholic beverages**	**0.01**	**-**	**-**	**0.01**	**-**
High alcohol content beverages	-	-	-	-	-
Low alcohol content beverages	0.01	-	-	0.01	-
**Appetizers**	**0.64**	**0.82**	**0.31**	**0.72**	**0.25**
**Cereals/Grains**	**0.37**	**0.40**	**0.26**	**0.41**	**0.10**
Grains and flours	0.14	0.21	0.10	0.15	0.06
Breakfast cereals and cereal bars	-	-	-	-	-
Bread	-	-	-	-	-
White bread	-	-	-	-	-
Brown bread	-	-	-	-	-
Others breads	-	-	-	-	-
Pasta	0.09	0.03	0.13	0.10	-
Bakery and pastry	0.14	0.16	0.03	0.16	0.05
**Eggs**	**-**	**-**	**-**	**-**	**-**
**Fish**	**-**	**-**	**-**	**-**	**-**
White fish	-	-	-	-	-

CAROTENES	Total 9–75	Children 9–12	Adolescents 13–17	Adults 18–64	Elderly 65–75
**(%)**	**2009**	**213**	**211**	**1655**	**206**
Oily fish	-	-	-	-	-
Shellfish	-	-	-	-	-
Canned fish	-	-	-	-	-
**Fruits**	**13.48**	**10.90**	**8.76**	**13.04**	**21.17**
**Meat and meat products**	**0.17**	**0.19**	**0.16**	**0.16**	**0.14**
Meat	-	-	-	-	-
Red meat	-	-	-	-	-
White meat	-	-	-	-	-
Poultry	-	-	-	-	-
Viscera and offal	0.05	0.08	-	0.06	-
Sausages and other meat products	0.12	0.10	0.16	0.10	0.14
**Milk and dairy products**	**7.53**	**11.15**	**12.58**	**7.45**	**4.69**
Milk	2.91	5.64	6.94	2.62	2.37
Whole milk	2.81	5.64	6.90	2.51	2.33
Semi skimmed milk	-	-	-	-	-
Skimmed milk	-	-	-	-	-
Other dairy	0.10	-	0.04	0.11	0.03

CAROTENES	Total 9–75	Children 9–12	Adolescents 13–17	Adults 18–64	Elderly 65–75
**(%)**	**2009**	**213**	**211**	**1655**	**206**
Yogurt and fermented milk	-	-	-	-	-
Skimmed Fermented milk	-	-	-	-	-
Whole fermented milk	-	-	-	-	-
Skimmed milk yogurts	-	-	-	-	-
Whole milk yogurt	-	-	-	-	-
Cheese	3.76	4.33	4.85	3.89	2.27
Other dairy products	0.85	1.19	0.78	0.93	0.05
**Non-alcoholic beverages**	**3.18**	**6.75**	**5.75**	**3.06**	**1.77**
Water	-	-	-	-	-
Coffee and herbal teas	-	-	-	-	-
Sugared soft drinks	-	-	-	-	-
Unsweetened soft drinks	-	-	-	-	-
Sports drinks	-	-	-	-	-
Juices and nectars	3.18	6.75	5.75	3.06	1.77
Energy drinks	-	-	-	-	-
Other non-alcoholic beverages	-	-	-	-	-
**Oils and fats**	**2.92**	**3.21**	**3.78**	**2.95**	**2.81**

CAROTENES	Total 9–75	Children 9–12	Adolescents 13–17	Adults 18–64	Elderly 65–75
**(%)**	**2009**	**213**	**211**	**1655**	**206**
Olive oil	1.28	1.47	1.43	1.32	0.98
Other oils	-	-	-	-	-
Butter, margarine and shortening	1.64	1.74	2.34	1.63	1.82
**Pulses**	**2.14**	**2.43**	**2.29**	**2.06**	**2.41**
**Sauces and condiments**	**8.35**	**12.14**	**13.92**	**8.32**	**3.68**
**Sugars and sweets**	**1.13**	**2.39**	**2.39**	**1.07**	**0.51**
Sugar	-	-	-	-	-
Chocolate	0.96	2.05	2.28	0.95	0.10
Jams and other	0.15	0.10	0.11	0.13	0.41
Other sweets	0.02	0.24	-	-	-
**Supplements and meal replacements**	**-**	**-**	**-**	**-**	**-**
**Ready-to-eat-meals**	**7.36**	**9.43**	**11.50**	**6.97**	**6.06**
**Vegetables**	**52.72**	**40.19**	**38.31**	**53.77**	**56.40**

Table S13. Dietary sources of vitamin E (%) from food groups/subgroups by sex and age groups in the ANIBES Spanish population.

VITAMIN E (%)	Total 9–75 2009	Children 9–12 213	Adolescents 13–17 211	Adults 18–64 1655	Elderly 65–75 206
**Alcoholic beverages**	**-**	**-**	**-**	**-**	**-**
High alcohol content beverages	-	-	-	-	-
Low alcohol content beverages	-	-	-	-	-
**Appetizers**	**0.95**	**1.58**	**0.89**	**0.99**	**0.50**
**Cereals/Grains**	**3.93**	**5.00**	**6.63**	**3.92**	**3.07**
Grains and flours	1.24	0.85	1.52	1.23	1.29
Breakfast cereals and cereal bars	0.18	0.02	0.11	0.24	0.18
Bread	0.57	0.31	0.28	0.59	0.61
White bread	-	-	-	-	-
Brown bread	0.23	0.10	0.08	0.24	0.34
Others breads	0.33	0.20	0.20	0.35	0.27
Pasta	0.05	-	0.05	0.05	-
Bakery and pastry	1.90	3.81	4.69	1.81	0.99
**Eggs**	**4.26**	**4.36**	**4.60**	**4.12**	**5.43**
**Fish**	**9.69**	**5.51**	**6.38**	**9.93**	**11.55**

VITAMIN E	Total 9–75	Children 9–12	Adolescents 13–17	Adults 18–64	Elderly 65–75
**(%)**	**2009**	**213**	**211**	**1655**	**206**
White fish	1.35	1.32	0.76	1.29	2.19
Oily fish	1.28	0.56	0.73	1.27	2.14
Shellfish	2.11	1.39	1.43	2.14	2.35
Canned fish	4.94	2.24	3.46	5.22	4.87
**Fruits**	**4.81**	**3.90**	**3.05**	**4.71**	**7.69**
**Meat and meat products**	**3.09**	**3.49**	**3.89**	**3.07**	**2.44**
Meat	1.64	1.61	1.83	1.64	1.45
Red meat	0.91	0.97	0.94	0.89	0.95
White meat	0.31	0.32	0.40	0.31	0.23
Poultry	0.41	0.33	0.49	0.44	0.27
Viscera and offal	0.06	0.03	-	0.06	0.08
Sausages and other meat products	1.40	1.85	2.06	1.37	0.91
**Milk and dairy products**	**4.38**	**5.39**	**5.10**	**4.44**	**3.70**
Milk	1.03	1.98	1.77	0.95	1.06
Whole milk	0.99	1.97	1.75	0.91	1.04
Semi skimmed milk	-	-	-	-	-
Skimmed milk	-	-	-	-	-

VITAMIN E (%)	Total 9–75 2009	Children 9–12 213	Adolescents 13–17 211	Adults 18–64 1655	Elderly 65–75 206
Other dairy	0.04	-	0.02	0.04	0.02
Yogurt and fermented milk	0.61	0.60	0.47	0.62	0.79
Skimmed Fermented milk	-	-	-	-	-
Whole fermented milk	0.11	0.01	0.04	0.10	0.21
Skimmed milk yogurts	0.02	-	0.01	0.02	0.02
Whole milk yogurt	0.49	0.58	0.43	0.50	0.56
Cheese	2.39	2.33	2.57	2.47	1.72
Other dairy products	0.36	0.49	0.28	0.40	0.12
**Non-alcoholic beverages**	**2.68**	**5.53**	**6.05**	**2.46**	**1.67**
Water	-	-	-	-	-
Coffee and herbal teas	-	-	-	-	-
Sugared soft drinks	-	-	-	-	-
Unsweetened soft drinks	-	-	-	-	-
Sports drinks	-	-	-	-	-
Juices and nectars	1.98	5.46	5.84	1.62	0.81
Energy drinks	-	-	-	-	-
Other non-alcoholic beverages	0.70	0.06	0.21	0.84	0.87

VITAMIN E	Total 9–75	Children 9–12	Adolescents 13–17	Adults 18–64	Elderly 65–75
**(%)**	**2009**	**213**	**211**	**1655**	**206**
**Oils and fats**	**45.66**	**47.92**	**43.44**	**45.83**	**43.36**
Olive oil	26.67	24.71	22.22	26.36	32.66
Other oils	16.77	20.73	19.34	17.30	7.78
Butter, margarine and shortening	2.22	2.48	1.88	2.17	2.92
**Pulses**	**1.72**	**1.59**	**1.48**	**1.66**	**1.90**
**Sauces and condiments**	**2.08**	**1.74**	**2.43**	**2.21**	**0.98**
**Sugars and sweets**	**0.70**	**1.66**	**1.40**	**0.66**	**0.18**
Sugar	-	-	-	-	-
Chocolate	0.63	1.61	1.28	0.57	0.18
Jams and other	-	-	-	-	-
Other sweets	0.07	0.05	0.12	0.09	-
**Supplements and meal replacements**	**0.18**	**0.21**	**-**	**0.22**	**-**
**Ready-to-eat-meals**	**4.44**	**5.74**	**7.22**	**4.29**	**2.45**
**Vegetables**	**11.42**	**6.41**	**7.44**	**11.48**	**15.06**

Table S14. Dietary sources of vitamin C (%) from food groups/subgroups by sex and age groups in the ANIBES Spanish population.

VITAMIN C	Total 9–75	Children 9–12	Adolescents 13–17	Adults 18–64	Elderly 65–75
**(%)**	**2009**	**213**	**211**	**1655**	**206**
**Alcoholic beverages**	**0.06**	**-**	**-**	**0.06**	**0.03**
High alcohol content beverages	-	-	-	-	-
Low alcohol content beverages	0.06	-	-	0.06	0.03
**Appetizers**	**0.30**	**0.41**	**0.57**	**0.30**	**0.04**
**Cereals/Grains**	**0.88**	**0.69**	**0.15**	**0.99**	**0.90**
Grains and flours	0.02	0.02	0.01	0.02	0.01
Breakfast cereals and cereal bars	0.86	0.67	0.14	0.96	0.89
Bread	-	-	-	-	-
White bread	-	-	-	-	-
Brown bread	-	-	-	-	-
Others breads	-	-	-	-	-
Pasta	-	-	-	-	-
Bakery and pastry	-	-	-	-	-
**Eggs**	**-**	**-**	**-**	**-**	**-**
**Fish**	**0.08**	**0.14**	**0.07**	**0.07**	**0.13**

VITAMIN C (%)	Total 9–75	Children 9–12	Adolescents 13–17	Adults 18–64	Elderly 65–75
	**2009**	**213**	**211**	**1655**	**206**
White fish	0.07	0.11	0.07	0.06	0.13
Oily fish	-	-	-	-	-
Shellfish	0.01	0.03	-	0.01	-
Canned fish	-	-	-	-	-
**Fruits**	**20.03**	**15.24**	**12.77**	**19.41**	**30.81**
**Meat and meat products**	**0.78**	**0.40**	**0.60**	**0.86**	**0.45**
Meat	0.54	0.35	0.60	0.58	0.21
Red meat	0.06	0.05	0.06	0.06	0.06
White meat	0.47	0.30	0.54	0.52	0.16
Poultry	-	-	-	0.01	-
Viscera and offal	0.25	0.05	-	0.27	0.24
Sausages and other meat products	-	-	-	-	-
**Milk and dairy products**	**8.93**	**14.70**	**13.29**	**8.59**	**6.46**
Milk	7.52	12.42	11.65	7.21	5.24
Whole milk	2.74	5.82	5.61	2.52	2.00
Semi skimmed milk	3.53	5.59	4.63	3.38	2.24
Skimmed milk	1.20	0.93	1.40	1.26	0.97

VITAMIN C	Total 9–75	Children 9–12	Adolescents 13–17	Adults 18–64	Elderly 65–75
**(%)**	**2009**	**213**	**211**	**1655**	**206**
Other dairy	0.05	0.08	0.01	0.05	0.03
Yogurt and fermented milk	0.94	1.03	0.64	0.93	1.13
Skimmed Fermented milk	-	-	-	-	-
Whole fermented milk	0.09	0.02	0.02	0.08	0.24
Skimmed milk yogurts	0.30	0.04	0.16	0.32	0.22
Whole milk yogurt	0.55	0.96	0.46	0.53	0.67
Cheese	0.01	0.01	0.01	0.02	0.01
Other dairy products	0.45	1.25	1.00	0.43	0.08
**Non-alcoholic beverages**	**8.56**	**15.64**	**12.68**	**8.58**	**5.24**
Water	-	-	-	-	-
Coffee and herbal teas	-	-	-	-	-
Sugared soft drinks	-	-	-	-	-
Unsweetened soft drinks	-	-	-	-	-
Sports drinks	0.47	0.99	0.39	0.57	0.07
Juices and nectars	8.07	14.66	12.29	7.99	5.18
Energy drinks	-	-	-	-	-
Other non-alcoholic beverages	0.02	-	-	0.02	-

VITAMIN C	Total 9–75	Children 9–12	Adolescents 13–17	Adults 18–64	Elderly 65–75
(%)	2009	213	211	1655	206
**Oils and fats**	**-**	**-**	**-**	**-**	**-**
Olive oil	-	-	-	-	-
Other oils	-	-	-	-	-
Butter, margarine and shortening	-	-	-	-	-
**Pulses**	**1.50**	**1.40**	**1.43**	**1.55**	**1.04**
**Sauces and condiments**	**3.03**	**4.27**	**4.74**	**3.13**	**0.84**
**Sugars and sweets**	**0.27**	**0.31**	**0.22**	**0.27**	**0.31**
Sugar	-	-	-	-	-
Chocolate	-	-	-	-	-
Jams and other	0.27	0.31	0.22	0.27	0.31
Other sweets	-	-	-	-	-
**Supplements and meal replacements**	**0.14**	**0.33**	**-**	**0.17**	**0.08**
**Ready-to-eat-meals**	**4.88**	**6.54**	**8.40**	**4.60**	**3.08**
**Vegetables**	**50.56**	**39.93**	**45.06**	**51.43**	**50.57**
